# Gut microbiota-derived ursodeoxycholic acid from neonatal dairy calves improves intestinal homeostasis and colitis to attenuate extended-spectrum β-lactamase-producing enteroaggregative *Escherichia coli* infection

**DOI:** 10.1186/s40168-022-01269-0

**Published:** 2022-05-28

**Authors:** Zhiyuan He, Yulin Ma, Sirui Yang, Shuyuan Zhang, Shuai Liu, Jianxin Xiao, Yajing Wang, Wei Wang, Hongjian Yang, Shengli Li, Zhijun Cao

**Affiliations:** State Key Laboratory of Animal Nutrition, College of Animal Science and Technology, China Agricultural University, Beijing, 100193 China

**Keywords:** Ursodeoxycholic acid, Enteroaggregative *Escherichia coli*, Extended-spectrum β-lactamase-producing *E. coli*, Neonatal dairy calf, Colitis, Hindgut microbiome, Fecal metabolome, Fecal microbiota transplantation

## Abstract

**Background:**

Antimicrobials are often used to prevent and treat diarrhea induced by enteroaggregative *Escherichia coli* (EAEC) in young ruminants. However, drug overuse or misuse accelerates the spread of multidrug-resistant extended-spectrum β-lactamase (ESBL)-producing *E. coli*. Thus, supplementary foods as alternatives to antibiotics are needed to prevent colibacillus diarrhea in neonatal dairy calves. Ursodeoxycholic acid (UDCA), a therapeutic bile acid, helps alleviate colitis. However, how UDCA helps alleviate ESBL-EAEC-induced clinical symptoms and colitis remains unclear.

**Results:**

We investigated the microbial profiles and metabolites of healthy and diarrheic neonatal calves to determine microbial and metabolite biomarkers in early-life development. Both the gut microbiota communities and their associated metabolites differed between healthy and diarrheic calves. Commensal *Butyricicoccus*, *Faecalibacterium*, *Ruminococcus*, *Collinsella*, and *Coriobacterium* were key microbial markers that distinguished healthy and diarrheic gut microbiomes. Random forest machine-learning algorithm and Spearman correlation results indicated that enriched UDCA, short-chain fatty acids (SCFAs), and other prebiotics were strongly positively correlated with these five bacterial genera. We explored the effect of ursodiol on bacterial growth, cell adherence, and lipopolysaccharide-treated Caco-2 cells. Adding ursodiol induced direct antibacterial effects, suppressed proinflammatory effects, and reduced cell integrity damage. Oral ursodiol delivery to neonatal mice exhibited significant antibacterial effects and helped maintain colonic barrier integrity in mouse models of peritonitis sepsis and oral infection. UDCA supplementation attenuated colitis and recovered colonic SCFA production. To validate this, we performed fecal microbiota transplantations to inoculate ESBL-EAEC-infected neonatal mice. Microbiotas from UDCA-treated neonatal mice ameliorated colitis and hindgut commensal bacterial damage compared with that of the microbiotas from the control and placebo mice, as evidenced by colonization of abundant bacteria, including Oscillospiraceae, Ruminococcaceae, Lachnospiraceae, and *Clostridia_UCG-014*, and upregulated SCFA production.

**Conclusions:**

This study provided the first evidence that UDCA could confer diarrhea resistance in ESBL-EAEC-infected newborn dairy calves. UDCA blocked bacterial growth and invasion both in vitro and in vivo, alleviated commensal bacterial dysbiosis during ESBL-EAEC infection in neonatal mouse models of sepsis and colitis via the TGR5-NF-κB axis, and upregulated SCFA production in the hindgut digesta. Our findings provide insight into the UDCA-mediated remission of ESBL-EAEC infections and the potential role of UDCA as an antibiotic alternative.

Video abstract

**Supplementary Information:**

The online version contains supplementary material available at 10.1186/s40168-022-01269-0.

## Background

Heifer breeding is important to both industry and academia and is closely related to the future productive potential of cattle herds. Heifers are susceptible to various bovine diseases of the immature gastrointestinal tract and immune system. Statistics on the morbidity of 450,000 heifers in China revealed that morbidity among suckling calves accounted for 51.4% of these deaths, and calf diarrhea accounted for 72.8% of the suckling-calf deaths (White Paper on China Dairy Replacement 2020). Thus, calf diarrheal prevention and timely treatment remain a great challenge. The causes of diarrhea in dairy calves include mainly pathogenic microorganisms such as *Escherichia coli*, Rotavirus, Coronavirus, and *Cryptosporidium*, and external factors such as nutrition, the environment, and feeding management. The morbidity and mortality rates from calf diarrhea due to diarrheagenic *E. coli* (DEC) are high and spreading fast among dairy calves, causing continuous problems for the dairy industry [1].

The clinical symptoms often manifest as diarrhea, toxemia, and colitis, further leading to secondary infection, growth retardation, and dysplasia post-DEC infection [2]. Recent progress has been made in research on the pathogenic mechanisms of DEC, and highly pathogenic plasmids or virulence genes carried by DEC usually encode specific adhesins, toxins, and siderophores, thus disrupting the host immune system [3]. Therefore, the primary task of prevention and control of extended-spectrum β-lactamase (ESBL)-producing enteroaggregative *Escherichia coli* (ESBL-EAEC) is to effectively prevent bacterial adhesion from the surfaces of intestinal epithelial cells, thus preventing bacterial colonization and invasion of virulence factors into the intestines. However, because of polymorphisms, understanding of ESBL-EAEC pathogenic mechanisms remains limited, especially of information on the whole genome and bacteria-host interaction mechanisms postinfection. Previous studies have shown that ESBL-EAEC infection causes major changes such as massive swelling, shedding, and epithelial barrier dysfunction [4], and especially bacterium-induced tissue inflammation, the key factor in colonic injury. Therefore, researchers must determine how to ameliorate the colonic inflammatory responses post-ESBL-EAEC infection.

Antimicrobials have been widely used as the main avenues for preventing and treating ESBL-EAEC infections. However, antibiotic abuse destroys diversity among the gastrointestinal flora, has long-term negative effects on human and animal healthcare, and induces rapid spread of multidrug-resistant bacteria [5, 6]. Therefore, safe and efficient antibiotic alternatives are urgently needed. As is known to all, animal bile is commonly used in traditional Chinese medicine and has important physiological significance and clinical value [7]. Bile acids constitute approximately 50% of the organic component of bile [8]. Once the primary bile acids, including cholic acid (CA) and chenodeoxycholic acid (CDCA) enter the gastrointestinal tract, over 50 natural secondary bile acids are produced [9]. Of these bile acids, the physiology and clinical applications of UDCA have been thoroughly summarized and analyzed in the literature. The Food and Drug Administration (FDA) has approved ursodiol, the most common formulation of UDCA. In fact, ursodiol is a typical “therapeutic” bile acid that serves as a biomarker of high-risk diseases and is widely used to treat primary sclerosing cholangitis and cholesterol gallstones [10–12] mainly owing to its anti-inflammatory, cytoprotective, and immunomodulatory effects [13]. Previous studies have confirmed that UDCA can alleviate dextran sulfate sodium-induced colitis and *Clostridioides difficile* infection (CDI) [14, 15]. However, the regulatory effects of UDCA on the community structure of the gut microbiota and whether UDCA can ameliorate the colonic inflammatory responses induced by ESBL-EAEC infection remain unclear.

Here, we used neonatal dairy calves as animal models to represent young ruminants. ESBL-EAEC infection altered the expression profiles of fecal metabolites, including the undermined UDCA in diarrheal feces. On the basis of existing theories and previous results, we speculated that UDCA plays a precise therapeutic role in alleviating the colonic inflammatory responses caused by ESBL-EAEC infection. We hypothesized that UDCA attenuates colitis mainly by blocking bacterial cell adherence and colonization inside the intestines, suppressing inflammation, and modulating the gut microbiota. We tested the alleviating effects of UDCA intervention in vitro and in Caco-2 cells with ESBL-EAEC-specific lipopolysaccharide (LPS)-induced inflammation. We then orally administered UDCA to mouse models of ESBL-EAEC-induced neonatal peritonitis sepsis and oral infection. Then, we detected the improved effects of the specific gut microbial communities from ursodiol-treated donors by comparing mice inoculated with ursodiol via fecal microbiota transplantation (FMT) with control mice. This study provides insight into the potential hazards and risks of ESBL-EAEC infection in young ruminants and suggests UDCA as a potential antibiotic alternative for future control of ESBL-EAEC infections in neonatal dairy calves.

## Methods

### Bacterial strains and cultures

The strain used in this study was isolated from an autochthonous clinical trial. Additional file 13: Table S1, and Additional file 14: Table S2, show the antibiotic susceptibility and genomic information of the strain [16]. *E. coli* K12 cells were preserved in our lab. Unless otherwise indicated, all bacterial strains were grown in Luria-Bertani (LB) broth (Qingdao Hope Biotechnology) or on MacConkey agar plates (Luqiao Company, Beijing, China) at 37 °C with shaking at 200 rpm. Antimicrobial drugs were purchased from the China Institute of Veterinary Drug Control. All *E. coli* isolates were screened for phenotypic identification of ESBL-producers on MacConkey agar containing cefotaxime (2 mg/L) and further confirmed using double-disc synergy testing in accordance with Clinical and Laboratory Standards Institute (CLSI) recommendations. Isolates were considered positive when the clear zone of inhibition of ceftazidime plus clavulanic acid or cefotaxime plus clavulanic acid was at least 5 mm larger than that of their respective single discs [17]. The culture was then diluted in fresh LB to a starting 600-nm optical density (OD_600_) of 0.2 in a 10-mL conical tube. Filter-sterilized ursodiol (0.03 and 0.3 g/L dissolved in dimethyl sulfoxide [DMSO]; Ursodiol USP, Spectrum Chemical, Shanghai, China, CAS 128-13-2) was added to each culture and incubated for 24 h at 37°C with shaking at 200 rpm. After mixing the cultures, the optical density was monitored at 4, 8, 12, and 24 h.

### Chemicals and reagents

Chemical reagents were obtained from Sigma-Aldrich (St. Louis, MO, USA), Steraloids Inc. (Newport, RI, USA), and TRC Chemicals (Toronto, ON, Canada). All standards were accurately weighed and prepared in water, methanol, sodium hydroxide solution, or hydrochloric acid solution to obtain individual stock solutions of 5.0 mg/mL. Appropriate amounts of each stock solution were mixed to create stock calibration solutions. Analytical-grade formic acid (Optima™ LC/MS grade) was obtained from Sigma-Aldrich. Methanol, acetonitrile, and isopropanol of Optima™ LC-MS grade were purchased from Thermo Fisher Scientific (Fair Lawn, NJ, USA). Ultrapure water was produced by a Mill-Q Reference system equipped with an LC-MS Pak filter (Millipore, Billerica, MA, USA). Enzyme-linked immunosorbent assay (ELISA) kits to test for Rotavirus, Coronavirus, and *Cryptosporidium* antigens in calf feces were obtained from IDEXX (Westbrook, ME, USA).

### Calf health status assessment and sample collection

Holstein newborn dairy calves were sourced from a conventional cow pasture in Heilongjiang Province, China, which feeds approximately 3173 heifers and 4945 adult cows and represents China’s typical dairy production practices. All animals used were antibiotic-free and individually examined to ensure that they were free of injury, disease, and dehydration. All calves were transferred to separate test sites after birth, and their conditions were appraised daily over the 21-day experimental period. The Beijing Association for Science and Technology (ID no. SYXK, 2016-0008) reviewed and approved the animal experimental protocols. The calves’ general appearance, rectal temperatures, fecal scores, and respiratory scores were recorded as previously described [18].

Briefly, 175 newborn female calves were included and fed 4 L of maternal colostrum from milking cows during the first 2 h of their lives. Calves were individually arranged in calf hutches to avoid direct contact and had free access to water. The calves were bucket-fed 4 L of milk replacer daily for 2–7 days (phase I) and 5 L for 8–14 days (phase II) after birth. The milk replacer contained 87.5% skim milk powder with 210 g/kg crude protein, 160 g/kg crude fat, 10 g/kg crude ash, and 19.2 MJ/kg metabolizable energy on a dry matter basis (Nutrifeed, IN, Netherlands). Diarrheic calves were observed for 2 consecutive days, during which they were fed normally without medical treatment. Those exhibiting diarrhea for at least 2 days were classified as “diarrheic” (defined as a fecal score ≥ 3 and infected with *E. coli* 1587) [1, 19]. Calves of the same age that never exhibited diarrhea were identified as “healthy” (defined as a fecal score ≤ 2 and without pathogen infection; Additional file 15: Table S3). No calves received antibiotics during the trial. Dedicated equipment and sterile gloves were used to collect rectal fecal samples to prevent cross-contamination and were allocated for detecting ESBL-EAEC strain 1587, Rotavirus, Coronavirus, and *Cryptosporidium* antigens via PCR or commercial ELISA kits. Finally, 63 neonatal calves were included with 15 healthy (H_1) and 15 diarrheic (D_1) calves in phase I, and 15 healthy (H_2) and 18 diarrheic (D_2) calves in phase II for the experimental period. All collected fecal samples (~ 10 g) were transferred directly to the laboratory on dry ice and stored at − 80 °C until used.

### Untargeted metabolomics analyses

Feces were thawed on an ice bath to reduce degradation. Approximately 5 mg of each lyophilized sample was weighed and transferred to a new 1.5-mL tube. Next, 25 μL of water was added, and the samples were homogenized with zirconium oxide beads for 3 min. Methanol (120 μL) containing internal standard was added to extract the metabolites. The samples were homogenized for another 3 min, then centrifuged at 1,800 g for 20 min, and 20 μL of supernatant was then transferred to a 96-well plate. The subsequent procedures were performed on an Eppendorf epMotion Workstation (Eppendorf Inc., Humburg, Germany). Freshly prepared derivative reagent (20 μL) was added to each well, then the plates were sealed and derivatized at 30 °C for 60 min. The samples were then further diluted by adding 330 μL of ice-cold 50% methanol solution, and the plates were stored at – 20 °C for 20 min, then centrifuged at 4000 g at 4 °C for 30 min. Next, 135 μL of supernatant was transferred to a new 96-well plate with 10 μL of internal standard per well. Serial dilutions of derivatized stock standards were added to the remaining wells. Finally, the plates were sealed for LC-MS analysis.

Metabolite profiling and data processing were performed using an ultra-performance liquid chromatography coupled to tandem mass spectrometry (UPLC-MS/MS) system (ACQUITY UPLC-Xevo TQ-S, Waters Corp., Milford, MA, USA). The analytes were separated on an ACQUITY UPLC BEH C18 1.7-μM VanGuard pre-column (2.1×5 mm) and analytical column (2.1×100 mm). Mobile phases were used as carried liquid at a constant flow rate of 0.4 mL/min. The source and desolvation temperatures were set at 150 °C and 500 °C, respectively. Each sample was analyzed via UPLC-MS/MS in both negative and positive ionization modes to acquire the metabolite profiles.

The raw data files generated by UPLC-MS/MS were processed using MassLynx software (version 4.1, Waters Corp.) to perform peak integration, calibration, and quantitation for each metabolite. The analysis order of all test samples was randomized. Quality control (QC) samples were obtained by mixing a small aliquot of each biological sample in the study set. The pooled QC samples represented both the sample matrix and metabolite compositions of the samples. The raw pooled QC mixtures were used to produce multiple QC samples that were analyzed during the whole injection sequence. In metabolomics, application of QC samples provides a mechanism to evaluate the quality and assess the analytical variance of the acquired data. The self-developed platform, iMAP (version 1.0, Metabo-Profile, Shanghai, China), was used for statistical analyses, including principal component analysis (PCA), partial least square-discriminant analysis (PLS-DA), univariate analysis, and pathway analysis.

### DNA extraction, PCR amplification, and 16S rRNA gene sequencing

Bacterial genomic DNA was extracted from fecal samples using QIAamp DNA Isolation Kits (Qiagen, Hilden, Germany). DNA concentration and integrity were assessed using a Nanodrop (Thermo Fisher Scientific) and 1.0% agarose gel electrophoresis. Amplification of the V3-V4 hypervariable region of the 16S rRNA gene was conducted via PCR using barcoded primers (338F: 5′-ACTCCTACGGGAGGCAGCAG-3′, 806R: 5′-GGACTACHVGGGTWTCTAAT -3′). Amplicon DNA at the optimal size (~ 450 bp) was purified from 1.2% agarose gel using a QIAquick PCR purification kit (Qiagen Science, Shanghai, China). The quality and quantity of the purified PCR products were checked using a Quant-iT PicoGreen dsDNA Assay Kit (Microplate reader, BioTek, FLx800) to ensure that all DNA concentrations were > 25 ng/μL. For the Illumina MiSeq sequencing, a PCR product library was prepared using the TruSeq Nano DNA LT Library Prep Kit (Illumina), then sequenced on the Illumina MiSeq platform (2 × 300, paired ends).

### Analysis of taxonomic and functional compositions

The main steps of the sequencing data analysis have been mentioned here. Sequencing data were processed using QIIME2, version 2020.02 [20]. The paired sequences were denoised, quality filtered, and merged using the DADA2 plugin (version 3.11) to obtain the amplicon sequence variants (ASVs) feature table [21]. Singleton ASVs and chimeric sequences were removed from further analyses. Taxonomic classification was performed using q2-feature-classifier (QIIME2 microbiome analysis platform). Finally, taxonomy was assigned to filtered ASVs using a pretrained QIIME2-compatible SILVA version 132 database, with 99% identity for the bacteria and representative sequences [22]. To determine the species diversity of each sample, alpha and beta diversity analyses were performed using q2-diversity in QIIME2 version 2020.02 (http://www.r-project.org/). To compare bacterial communities between individuals and groups, Jaccard, Bray-Curtis, unweighted UniFrac and weighted UniFrac outputs were assessed and visualized using unsupervised PCoA analysis. Differences between groups were detected using PERMANOVA in the “vegan” package in R software (version 3.3.1). Treatment-dependent features were identified using LEfSe 1.0 (http://huttenhower.sph.harvard.edu/galaxy/root?tool_id=lefse_upload) [23]. A size-effect threshold of 4 on the logarithmic LDA score was used to identify discriminating taxa. PICRUSt analysis was used to predict gene family abundances of the gut microbial communities based on the 16S rRNA gene composition. The constructed ASV feature table was converted to PICRUSt format and normalized to 16S rRNA gene copy numbers to correct for over- and underestimation of microbial abundances. The normalized dataset was analyzed using the Kyoto Encyclopedia of Genes and Genomes (KEGG) dataset.

### Cell cultures and treatments

The human colonic adenocarcinoma cell line, Caco-2 (ATCC-HTB-37, Manassas, VA, USA) was maintained in modified Eagle’s medium (MEM; Gibco, NY, USA) supplemented with 10% fetal bovine serum (Gibco) and 1× penicillin-streptomycin-L-glutamine (Gibco, 10378-016) at 37 °C in a 5% CO_2_ incubator. For the adhesion assays, cells were grown in 24-well tissue culture plates until reaching 80% confluence, then incubated with *E. coli* strains at a multiplicity of infection of 10 after washing in MEM. Cell-associated bacteria were quantified by removing nonadhering bacteria, which was performed after washing and cell lysis. Colony-forming units (CFUs) were counted via serial dilution, and the adherence percentage was calculated by dividing the final CFU/mL count by the initial CFU/mL count for each replicate.

To investigate the effect of LPS on Caco-2 cells, purified LPS component was isolated from *E. coli* 1587 using an LPS isolation kit (Sigma, MAK339) and diluted in MEM to a storage concentration of 1 mg/mL. To evaluate the effects of UDCA on LPS stimulation, ursodiol dissolved in DMSO (0.15 g/L) or the same volume of DMSO was added to MEM, and the cells were cultured for 12 h. LPS (10 μg/mL final concentration) was then added to this culture medium, and the cells were further cultured for 4 h.

### Murine treatment and sample collection

Littermate pregnant specific pathogen-free (SPF) CD-1 mice (Sipeifu Biotechnology, Beijing, China) were adapted to standardized environmental conditions (temperature: 25 ± 2 °C; humidity: 55 ± 10%), with a 12-h/12-h light/dark cycle. Mice were maintained in strict accordance with the rules of the Administration of Affairs Concerning Experimental Animals approved by the State Council of the People’s Republic of China (14-11-1988). For neonatal mice, the day of birth was considered day 0, and female mice were screened on day 2 and verified to have ingested breast milk (abdominal milk spot). After 1 week of acclimatization, infection tests were performed via intraperitoneal injection or oral gavage with a sublethal dose of *E. coli* 1587 persisters suspension (3.0 × 10^5^ CFU/mouse). Mice were weaned on day 21.

Two-day-old female mice were randomly divided into three treatment groups with twelve mice and one dam per group and were orally gavaged daily with 50 μL corn oil containing 70 mg/kg body weight of ursodiol or the same volume of corn oil alone for 14 days. Neonatal mice were concurrently challenged with or without *E. coli* 1587 via intraperitoneal injection on day 0. The treatment groups were as follows: (1) control group: free access to regular sterile water for 2 weeks without infection; (2) placebo group: daily gavage of corn oil with regular sterile water for 2 weeks with infection; and (3) ursodiol group: daily gavage of corn oil with ursodiol for 2 weeks with infection. From days 0 to 7, body weight was measured daily, and the disease activity index (DAI) was evaluated to assess the colitis severity as previously described [24]. The mice were euthanized by anesthesia on days 3 and 7. Serum was separated from the eyeball blood by standing overnight at 4 °C, then stored at − 80 °C. Colon lengths were measured, and a 5-mm segment of hollow mid-colon was fixed in 10% formalin for subsequent histological analysis. The remaining colon and colonic contents were collected aseptically from each mouse and snap-frozen in liquid nitrogen for further analysis. Colonic tissues were homogenized in sterile phosphate-buffered saline (PBS) to count bacterial loads using a CFU calculation method.

To study the effect of UDCA in the mouse model of oral infection, another test was conducted using 2-day-old female mice. The mice were randomly divided into four groups with six mice and one dam per group and were orally gavaged daily with 50 μL corn oil containing 70 mg/kg body weight of ursodiol for 14 days. Mice were simultaneously challenged with *E. coli* 1587 via oral gavage on day 0 to induce colitis. The treatment groups were as follows: (1) control group: daily gavage of corn oil with regular sterile water for 2 weeks without infection; (2) ursodiol group: daily gavage of corn oil with ursodiol for 2 weeks without infection; (3) 1587 group: daily gavage of corn oil with regular sterile water for 2 weeks with infection; and (4) 1587*+*ursodiol group: daily gavage of corn oil with ursodiol for 2 weeks with infection. From days 0 to 7, body weight and DAI were measured daily. All mice were euthanized with anesthesia on day 7. Colon lengths were measured, and a 5-mm segment of hollow mid-colon was fixed in 10% formalin for subsequent histological analysis. The remaining colon and colonic contents were collected aseptically from each mouse and snap-frozen in liquid nitrogen for subsequent analyses. Colonic tissues were homogenized in sterile PBS to count bacterial loads. Serum was prepared from the eyeball blood by standing overnight at 4 °C, then frozen at − 80 °C to detect serum cytokine concentrations.

For the fecal transplantations, 2-day-old female mice were randomly divided into three treatments with four mice and one dam per group and orally gavaged daily with 50 μL corn oil containing 70 mg/kg body weight of ursodiol or the same volume of corn oil alone for 14 days. Mice were concurrently challenged with *E. coli* 1587 via oral gavage on day 0. The treatment groups were as follows: (1) control group: free access to regular sterile water for 2 weeks without infection; (2) placebo group: daily gavage of corn oil with regular sterile water for 2 weeks with infection; and (3) ursodiol group: daily gavage of corn oil with ursodiol for 2 weeks with infection. On day 7, fresh feces were aseptically collected from all three groups for FMT. Fresh feces collected separately were homogenized in sterile saline to a final concentration of 100 mg feces/mL. Pooled samples were centrifuged at 500 rpm for 5 min, and the supernatant was filtered through 100-μm filters, then used for FMT treatment. Two-day-old female mice were randomly divided into three treatment groups with six mice and one dam per group, and 50 μL of FMT was administered per mouse via oral gavage twice daily for 1 week. Mice were again challenged with *E. coli* 1587 via oral gavage on day 8. From days 8 to 14, body weight and DAI were measured daily, and fresh feces were collected from each mouse on days 14 and 15 to measure the fecal microbiotas. All mice were euthanized by anesthesia on day 15, and colon lengths were measured. Colonic contents were collected aseptically from each mouse and snap-frozen in liquid nitrogen for subsequent analyses.

### RNA extraction and quantitative reverse transcription-PCR analysis

Caco-2 cells were harvested, and frozen colon tissues were obtained from each group of mice after euthanasia. Whole-cell or tissue RNA was extracted using the EASYspin Plus kit (Aidlab, Beijing, China) per the manufacturer’s instructions. Total RNA was treated with DNaseΙ, and 500 ng of total RNA was used for cDNA synthesis by reverse transcription using the PrimeScript RT reagent kit with gDNA Eraser (Takara, Beijing, China). The specific primers for interleukin (IL)-1β (5′-ACGATGCACCTGTACGATCACT-3′; 5′-CACCAAGCTTTTTTGCTGTGAGT-3′), IL-6 (5′-GCTGCAGGCACAGAACCA-3′; 5′-TAAAGTGCGCAGAATGAGATG-3′), IL-10 (5′-GCTGGAGGACTTTAAGGGTTACCT-3′; 5′-CTTGATGTCTGGGTCTTGGTTCT-3′), TNF-α (5′-TGCTCCTCACCCACACCAT-3′; 5′-GGAGGTTGACCTTGGTCTGGTA-3′), TGF-β (5′-GCGCATCCTAGACCCTTTCTC-3′; 5′-CAGAAGGTGGGTGGTCTTGAA-3′), TGR5 (5′-TCCTGTCAGTCTTGGCCTATGA-3′; 5′-GGTGCTGCCCAATGAGATG-3′), and GAPDH (5′-GAAGGTGAAGGTCGGAGTC-3′; 5′-GAAGATGGTGATGGGATTTC-3′) were designed as previously described [25–27]. Real-time PCR was performed, and the products were detected using QuantStudio 6 Flex Real-Time PCR (Applied Biosystems, Foster City, CA, USA). PCR was performed in a 20-μL volume containing 1 μL cDNA, 10 μL 2×SYBR green Premix Ex Taq (TaKaRa Bio Inc., Japan), and 0.4 μM of each gene-specific primer. Thermal cycling parameters were 95 °C for 2 min, followed by 42 cycles of 95 °C for 15 s, 60 °C for 15 s, and 72 °C for 15 s, and 1 cycle of 95 °C for 30 s, 60 °C for 30 s, and 95 °C for 30 s. Finally, a melting curve was obtained for the PCR product to determine the amplification specificity. Each sample was run in triplicate on the 96-well sample plate, and each gene expression level was calculated relative to the GAPDH gene expression.

### Western blot analysis

After the treatments, a portion of the frozen colon samples or whole cells were homogenized and lysed with lysis buffer (50 mM Tris-HCl, pH 7.4, 150 mM NaCl, 1% Triton X-100, 5 mM MgCl_2_, 10% glycerol, 1 mM EDTA, and 1× protease inhibitor cocktail C). Cell debris was removed by centrifugation at 12,000 × g for 10 min at 4 °C. Protein contents were quantified using a bicinchoninic acid (BCA) protein assay kit (Beyotime Biotechnology, Beijing, China) following the manufacturer’s instructions. All samples were fractionated by 10–12% sodium dodecyl sulfate-polyacrylamide gel electrophoresis (SDS-PAGE) gels and transferred onto a polyvinylidene difluoride membrane. After blocking with 5% skim milk, the membranes were incubated with the indicated antibodies, followed by an appropriate horseradish peroxidase (HRP)-conjugated secondary antibody. Blots were visualized using an enhanced chemiluminescence kit (Beyotime Biotechnology, Beijing, China). The following antibodies were used in the western blot assays: HRP-conjugated goat anti-mouse and anti-rabbit IgG (DingGuo; Beijing, China), β-actin (ABclonal Technolog, AC004), phospho-IκBα (Ser36) (Abcam, ab133462), IκBα (Abcam, ab32518), and occludin (ABclonal Technolog, A2601).

### Quantification of serum inflammatory cytokines

Murine blood samples were allowed to stand overnight, then the serum samples were acquired via centrifugation at 5000 rpm for 10 min. Concentrations of inflammatory cytokines, including IL-1β and IL-10, in the serum samples were measured using ELISA kits (ABclonal Technology, Wuhan, China) per the manufacturer’s instructions.

### Histological analysis

For morphological measurements, colonic tissues from the euthanized mice were fixed in 10% formalin, routinely processed, and embedded in paraffin. Next, 4-μm-thick sections were cut and stained with hematoxylin and eosin. The images were analyzed using ImageJ software (version 1.8.0_172, National Institutes of Health, Bethesda, MD, USA). The ulcers, ulceration, crypt structure, crypt loss, and presence or absence of edema were measured, and the histological score was determined as previously described [28].

### Quantification of SCFA profiles

SCFAs (acetate, propionate, and butyrate) in the colonic contents or fecal samples were quantified with gas chromatography. Briefly, 100 mg of samples were dissolved, homogenized, and centrifuged at 5400 rpm for 10 min. The supernatant was mixed with 0.2 mL deproteinized solution of 2-ethylbutyric acid in an ice-water bath for 30 min, then recentrifuged at 10,000 rpm for 10 min. The acquired supernatant was then kept in a 2-mL screw-cap vial and analyzed for the SCFA content using a gas chromatography system (Agilent Technologies, Wilmington, DE, USA).

### Statistical analysis

Distances in the coefficients of variation of the relative fecal genus abundances in the calf feces and predicted genes at each day of age between the healthy and diarrheic calves were assessed using the nonparametric Kruskal-Wallis test, and multiple comparisons were conducted using the Mann-Whitney-Wilcoxon *U* test. Significant differences were declared after interpretation and visualization. For many metabolomics studies, multivariate statistical analyses (e.g., PCA, PLS-DA, orthogonal partial least square discriminant analysis [OPLS-DA], and random forest analysis) and univariate statistical analyses (e.g., the nonparametric Kruskal-Wallis test, Student’s *t*-test, the Mann-Whitney-Wilcoxon *U* test, analysis of variance [ANOVA], and correlation analysis) are extensively performed. Statistical algorithms were adapted from the widely used statistical analysis software packages in R studio (http://cran.r-project.org/). The statistical methods were determined based on the data and project goals. All data are presented as the mean ± standard error of the mean (SEM). Statistical analyses were conducted using GraphPad Prism 5.0 (Version 5.2.1350.0) and SPASS software (version 19.0). *P*-values were determined using unpaired *t*-tests between two groups, two-way ANOVA among various treatment groups, and the two-sided, log-rank (Mantel-Cox) test. Statistical differences were determined at *P < 0.05, **P < 0.01, and ***P < 0.001. All experimental data were representative of three independent experiments with similar results.

## Results

### ESBL-EAEC infection induced temporal changes in the hindgut microbiota

**Fig. 1:**
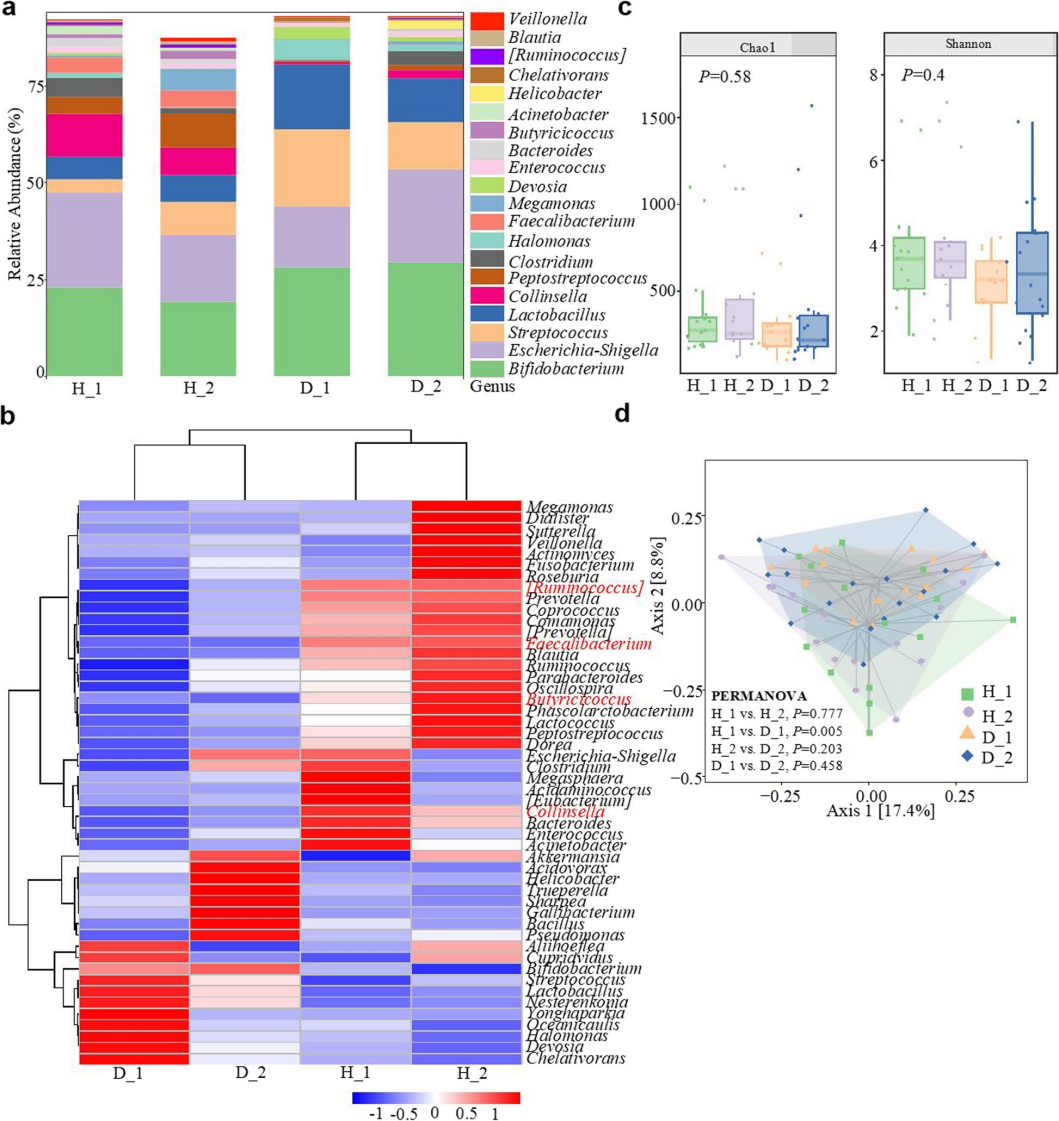
Effect of ESBL-EAEC infection-driven gut microbiota assembly in diarrheic calves. **a** Relative abundances of fecal bacterial genera in 99.5% of the community. **b** Heat map of the top 50 bacterial genera in fecal samples from diarrheic (D) or healthy (H) calves. Color indicates the relative bacterial abundances in the group samples; the corresponding relationship between the color gradient and the value is shown in the gradient color block. *Ruminococcus*, *Butyricicoccus*, *Faecalibacterium*, and *Collinsella* are in red. **c** α-Diversity of different groups as per the Chao1 or Shannon index. Data are presented as the mean ± SEM. *P*-values were determined using the nonparametric Kruskal-Wallis test. **d** Principal coordinate analysis (PCoA) based on the weighted UniFrac distance matrix. Data were analyzed using PERMANOVA, with 999 permutations

The causes of diarrhea in dairy calves are complex, including both pathogens and external factors. DEC infections lead to high morbidity and mortality rates in diarrheic calves, and DEC disseminates quickly among neonatal dairy calves, causing continuous hazards to the dairy industry [1]. To gain insight into the underlying regulatory mechanisms of calves during natural ESBL-EAEC infections, we collected fecal samples from healthy and diarrheic calves in two phases and performed multi-omics analyses (Additional file 15: Table S3). We assessed the gut microbial profiles of healthy (*n* = 15, phase I; *n* = 15, phase II) and diarrheal calves (*n* = 15, phase I; *n* = 18, phase II) using metataxonomic methods. Analysis of 16S ribosomal RNA (rRNA) genes of the fecal microbiota demonstrated that Firmicutes, Actinobacteria, Proteobacteria, Bacteroidetes, and Fusobacteria were predominant phyla in all fecal microbiotas, and Proteobacteria was notably increased in the D_2 group (Additional file 1: Fig. S1a). At the family level, bacterial compositions in the diarrheic calves were enriched with Streptococcaceae, Lactobacillaceae, and Bifidobacteriaceae, but lacked Coriobacteriaceae, Peptostreptococcaceae, Ruminococcaceae, and Lachnospiraceae (Additional file 1: Fig. S1b). At the genus level, 50 genera were considerably differentiated among the healthy and diarrheal calves, particularly *Butyricicoccus*, *Faecalibacterium*, *Collinsella*, and *Ruminococcus* (Fig. 1a, b). Alpha diversity was tested with the Chao1 and Shannon indexes between groups. No significant differences were noted between the healthy and diarrheic calves; however, the gut microbiota diversity showed a strong tendency to decline in the diarrheic calves (Fig. 1c). Principal co-ordinates analysis (PCoA) based on weighted UniFrac distance yielded dispersed data points on the plots of the healthy and diarrheal calves, implying microbial dysbiosis in the guts of the diarrheic calves, especially between the H_1 and D_1 groups (*P* = 0.005; Fig. 1d). Differentially abundant fecal bacterial taxa were further identified by LEfSe analysis. *Faecalibacterium*, *Butyricicoccus*, and *Collinsella* were abundant in the H_1 calves, and *Faecalibacterium*, *Butyricicoccus*, *Ruminococcus*, and *Coriobacterium* differentiated the H_2 group (Additional file 1: Fig. S1c, d). KEGG enrichment pathway analysis indicated that differentially expressed genes (DEGs) from the H_1 vs D_1 and H_2 vs D_2 groups were abundant in several general pathways, including infectious diseases, amino acids, carbohydrates, lipid metabolism, and biosynthesis of other secondary metabolites (Additional file 2: Fig. S2). These results indicate that in the diarrheic neonatal calves, ESBL-EAEC induction strongly influenced the gut microbiota structure, particularly for the commensal bacteria, Coriobacteriaceae, Ruminococcaceae, and Lachnospiraceae, in which *Faecalibacterium*, *Butyricicoccus*, and *Ruminococcus* are typical butyrate-producing bacteria in mammals [29] and further affected the bacterial metabolism as indicated above.

### Gut microbiota-derived UDCA served as a key metabolite conferring resistance against ESBL-EAEC-induced diarrhea in neonatal dairy calves

**Fig. 2:**
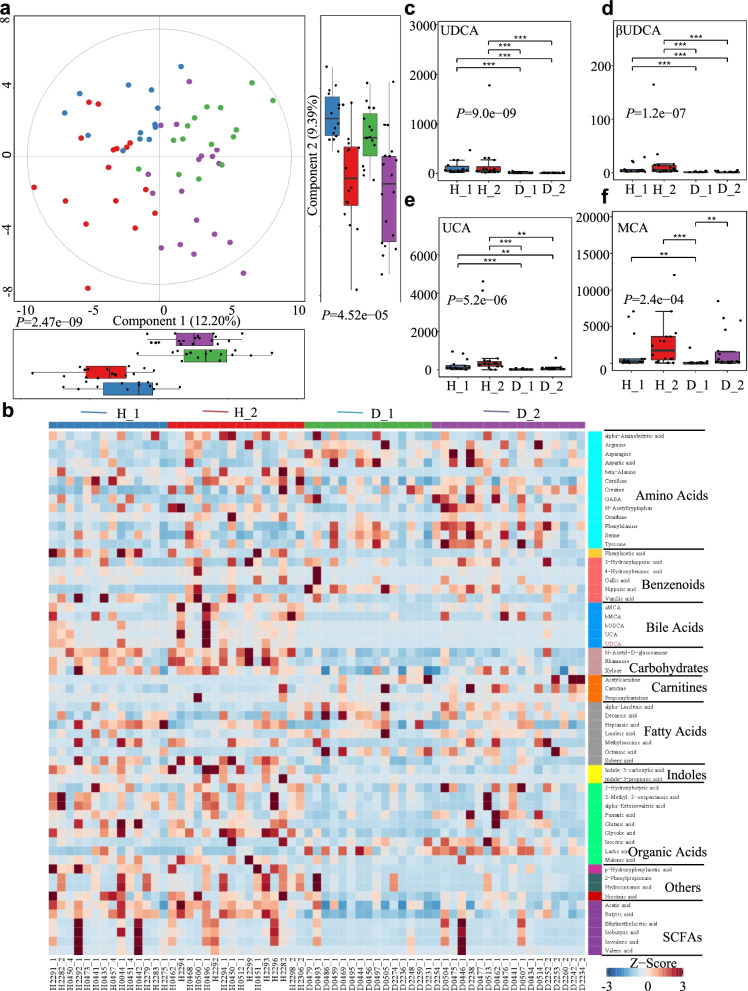
Changes in the fecal metabolomic profiles of the diarrheic calves. **a** The fecal metabolomic profiles were clustered using PLS-DA. The metabolomic profiles for the H_1, H_2, D_1 and D_2 groups are shown in the same colors. Data are presented as the mean ± SEM. *P-*values were determined using the nonparametric Kruskal-Wallis test. **b** Relative abundances of metabolites were clustered using a UPGMA dendrogram and shown in a heatmap. Color indicates the relative abundances of the metabolite in the group of samples; the corresponding relationship between the color gradient and the value is shown in the gradient color block. The metabolite variation is shown using Z_Score. UDCA is in red. The concentrations of fecal UDCA (**c**), β-UDCA (**d**), UCA (**e**), and MCA (**f**) are displayed as box and dot plots. Data are presented as the mean ± SEM. *P*-values were determined using the nonparametric Kruskal-Wallis test. *P ≤ 0.05, **P ≤ 0.01, ***P ≤ 0.001

Understanding how gut microorganisms and microbial-derived products affect the incidence of calf diarrhea is vital [30]. To evaluate the effects of gut microbiota alterations on the intestinal environment, we used untargeted metabolomics to study the changes in fecal metabolomes of healthy (*n* = 30) and diarrheic calves (*n* = 33) using UPLC-MS/MS. Metabolite classification indicated that 24.43% of the compounds were amino acids, 11.45% were bile acids, and 6.87% were SCFAs (Additional file 3: Fig. S3a). Similar to the gut microbial metataxonomic analyses, PLS-DA revealed widely dispersed data points on the plots by the experimental group of the healthy fecal metabolomes from the diarrheal samples (Component 1, *P* = 2.47e−09; Component 2, *P* = 4.52e−05; Fig. 2a, Additional file 3: Fig. S3b). PCA analyses based on the Bray-Curtis distance showed similar separation in the metabolite structures between the healthy and diarrheal fecal metabolomes (PC1, *P* = 0.001; PC2, *P* = 0.045; Additional file 3: Fig. S3c, d). Moreover, PLS-DA analyses showed a separation of the metabolites between H_1 and D_1, H_2 and D_2, H_1 and H_2, and D_1 and D_2 groups (Additional file 4: Fig. S4), indicating that the fecal metabolomes were closely related to the ESBL-EAEC infection stage or process.

**Fig. 3:**
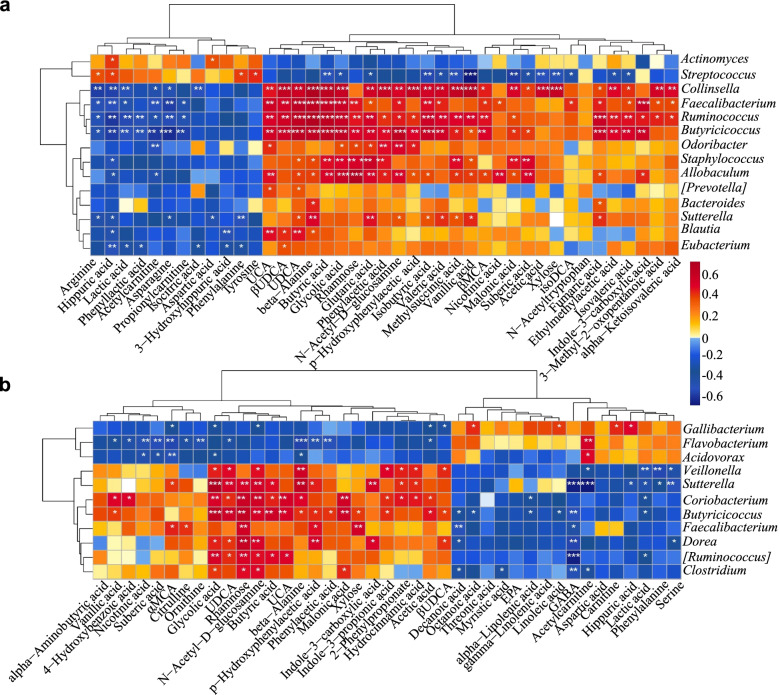
Spearman correlation between intestinal microbiotas and metabolites in H_1 vs D_1 (**a**); H_2 vs D_2 (**b**). Red denotes a positive correlation; blue denotes a negative correlation. The color intensity is proportional to the strength of the Spearman correlation. *P ≤ 0.05, **P ≤ 0.01, ***P ≤ 0.001

The relative quantities of fecal metabolites in both healthy and diarrheic calves were visualized using a heatmap, and the top 58 metabolites are listed and clustered in Fig. 2b and Additional file 16: Table S4. The concentrations of several bile acids (UDCA, βUDCA, UCA, and MCA) were higher in the healthy calves than in the corresponding diarrheic calves (Fig. 2c–f), and the UDCA concentration was dramatically enriched in the healthy calves (*P* = 9.0e−09). The relative fold changes and variations in fecal metabolites were visualized using volcano plots for the H_1 vs D_1, H_2 vs D_2, H_1 vs H_2, and D_1 vs D_2 groups, and UDCA was substantially downregulated in both the D_1 and D_2 groups (Additional file 5: Fig. S5). KEGG pathway analyses demonstrated that these differential metabolites were mostly concentrated in organic-acid-related metabolisms, including arginine and proline metabolism; phenylalanine, tyrosine, and tryptophan biosynthesis; bile acid biosynthesis; and linoleic acid metabolism (Additional file 3: Fig. S3e). We therefore further analyzed the fecal metabolite profiles using a random forest supervised machine-learning algorithm to construct the model for diagnosing diarrhea in early-life dairy calves. Threefold cross-validation results revealed that 10 of 58 fecal metabolites, including UDCA, βUDCA, αMCA, βMCA, and UCA, mostly contributed to the discrimination power of the dairy calf health status, and hippuric acid was a biomarker of diarrhea status. The relative abundance ranks of those 10 metabolite markers were plotted against the healthy status, represented by the MeanDecreaseGini score (Additional file 3: Fig. S3f; Additional file 17: Table S5). Gut microbes produce secondary bile acids by deconjugating host-derived primary bile acids via bile salt hydrolases possessed by three major phyla (Firmicutes, 30%; Bacteroidetes, 14.4% and Actinobacteria, 8.9%) [31]. Within these phyla, *Clostridium*, *Ruminococcus*, and *Collinsella* are the main producers of UDCA and mediate the epimerization reaction from CDCA to UDCA via 7α/7β-hydroxysteroid dehydrogenases [32–34]. Spearman correlation analysis was performed to interpret the relevance between differentially enriched bacteria and heterogeneous metabolites. H_1-enriched UDCA was strongly positively correlated with *Collinsella* (*R* > 0.57, *P* = 0.0011), *Faecalibacterium* (*R* > 0.64, *P* = 0.00012), *Ruminococcus* (*R* > 0.63, *P* = 0.00015), *Butyricicoccus* (*R* > 0.66, *P* = 6.68e−05), and *Blautia* (*R* > 0.47, *P* = 0.0079; Fig. 3a). Similarly, H_2-enriched UDCA was significantly positively correlated with *Sutterella* (*R* > 0.46, *P* = 0.0069), *Coriobacterium* (*R* > 0.37, *P* = 0.031), *Butyricicoccus* (*R* > 0.48, *P* = 0.0038), and *Ruminococcus* (*R* > 0.37, *P* = 0.032; Fig. 3b). Importantly, these microbes were also positively correlated with upregulated bile acids, SCFAs, indoles, and carbohydrates and negatively correlated with amino acid consumption, increased hippuric acid and lactic acid. Additionally, *Streptococcus* was strongly correlated with enriched hippuric acid (*R* > 0.40, *P* = 0.028). Thus, the reduced UDCA content was likely due to decreased Coriobacteriaceae, Ruminococcaceae, and Lachnospiraceae abundances in the healthy calves. Taken together, these data suggest that the ESBL-EAEC-induced diarrhea was accompanied by changes in bile acid metabolism or bile acid pools and thus suppressed UDCA biosynthesis and impaired the commensal bacterial structure. Our findings also implied that UDCA supplementation might directly improve intestinal homeostasis through specific modes of action.

### Antibacterial effects of ursodiol and suppression of specific LPS-induced colonic cell inflammation

**Fig. 4:**
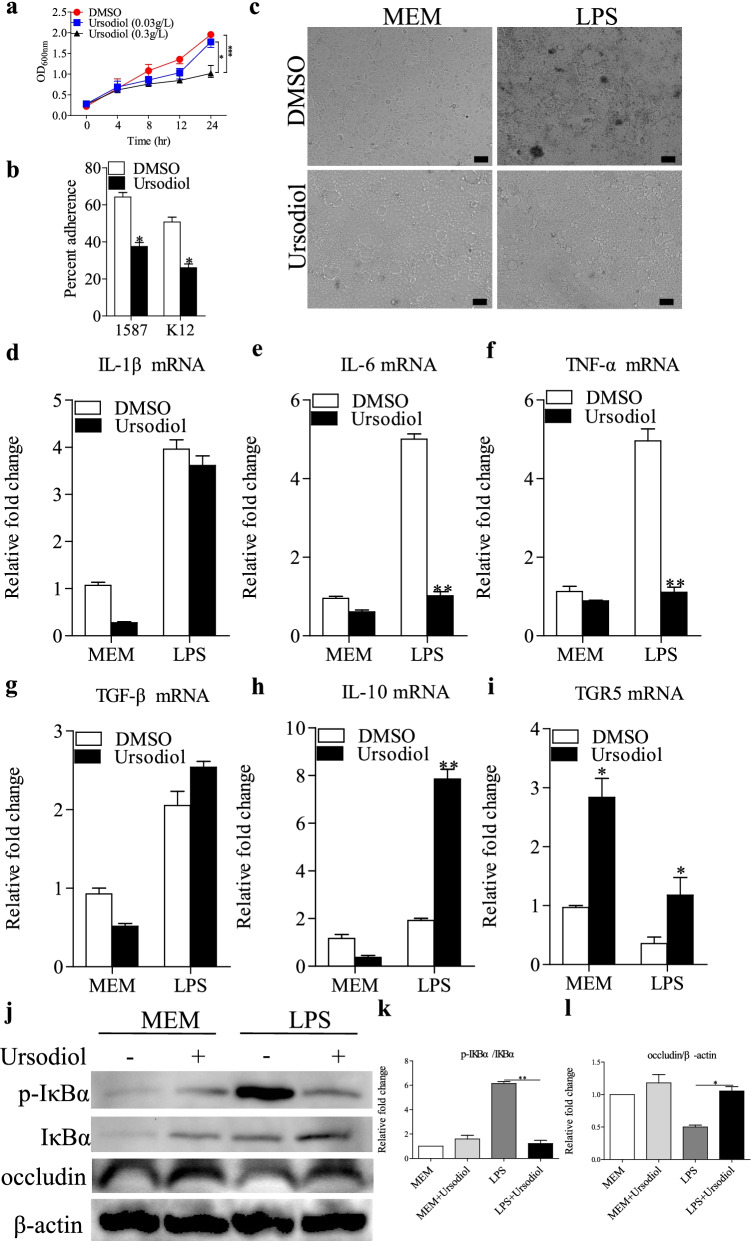
Antibacterial effects of ursodiol on ESBL-EAEC and suppression of specific LPS-induced inflammation in Caco-2 cells. **a** Ursodiol inhibited ESBL-EAEC strain growth in vitro. Growth kinetics of *E. coli* 1587 over a 24-h culture period. Growth curves were acquired in LB medium with DMSO and addition of different concentrations of ursodiol. Data are presented as means ± SEM from triplicate experiments. Statistical significance was determined using two-way ANOVA. *P ≤ 0.05, **P ≤ 0.01, ***P ≤ 0.001 relative to the ursodiol-treated (0.3 g/L) group. **b** Ursodiol administration blocked cell adherence of *E. coli* 1587 and *E. coli* K12. **c** Morphology of Caco-2 cells treated with LPS component extracted from strain 1587. Relative mRNA expression levels of five representative inflammatory cytokines and TGR5, IL-1β (**d**), IL-6 (**e**), TNF-α (**f**), TGF-β (**g**), IL-10 (**h**), TGR5 (**i**) in the cells. **j** Western blot analysis of NF-κB pathway activation and cell tight junctions. Relative fold changes of p-IκBα (**k**) and occludin (**l**) are presented as means±SEM. Statistical significance was analyzed using unpaired *t*-tests. *P ≤ 0.05, **P ≤ 0.01, ***P ≤ 0.001. All experimental data are representative of three independent experiments with similar results

We performed in vitro assays to assess bacterial growth to determine the direct effects of ursodiol on the life cycle of clinically relevant ESBL-EAEC 1587. *E. coli* 1587 growth decreased significantly and dose-dependently when exposed to ursodiol at 8, 12, and 24 h compared with the DMSO-treated control group (Fig. 4a). Besides, *E. coli* 1587 isolates adhered significantly better than did a laboratory *E. coli* K12 strain when added to Caco-2 cell cultures, and adding ursodiol markedly mitigated cell adherence of both strains, indicating that ursodiol directly blocked bacterial adhesion from the intestinal epithelial cell surface (Fig. 4b).

LPS activity is closely related to *E. coli* pathogenicity, and therefore, purified LPS component was separated from *E. coli* 1587 and added to Caco-2 cells pretreated with optimal concentrations of ursodiol or DMSO. Adding ursodiol rescued the LPS-induced morphological changes, such as shrinking and detaching, in the Caco-2 cells (Fig. 4c), which were harvested and subjected to real-time PCR analyses. Ursodiol decreased the mRNA expression levels of proinflammatory cytokines IL-6 and TNF-α after LPS treatment, while the anti-inflammatory cytokine IL-10 was upregulated (Fig. 4d–h). Importantly, ursodiol exposure significantly increased TGR5 transcription levels in Caco-2 cells after these levels were downregulated by LPS stimulation (Fig. 4i).

Disruption of the gut mucosal barrier and bacterial invasion promotes intestinal inflammation mainly via TLR4/NF-κB pathway stimulation in intestinal epithelial cells [35], and TGR5, a membrane-bound bile acid receptor, antagonizes gastric inflammation partly by inhibiting NF-κB signaling [27]. We detected relative phosphorylation levels of IκBα between the treatment groups via western blot assay. Cotreatment with ursodiol markedly blocked the LPS-induced increases in IκBα phosphorylation levels (Fig. 4j, k). LPS treatment significantly reduced the cell tight-junction-related protein, occludin, but ursodiol pretreatment countered this downregulation (Fig. 4j, l). These results demonstrated that the antibacterial effects of ursodiol administration were mainly exerted by blocking bacterial growth and cell adherence in vitro. Ursodiol exposure significantly increased TGR5 transcription levels in Caco-2 cells, thus mitigating NF-κB signaling activation.

### Oral ursodiol alleviated ESBL-EAEC-induced colitis in a neonatal mouse model of peritonitis sepsis

**Fig. 5:**
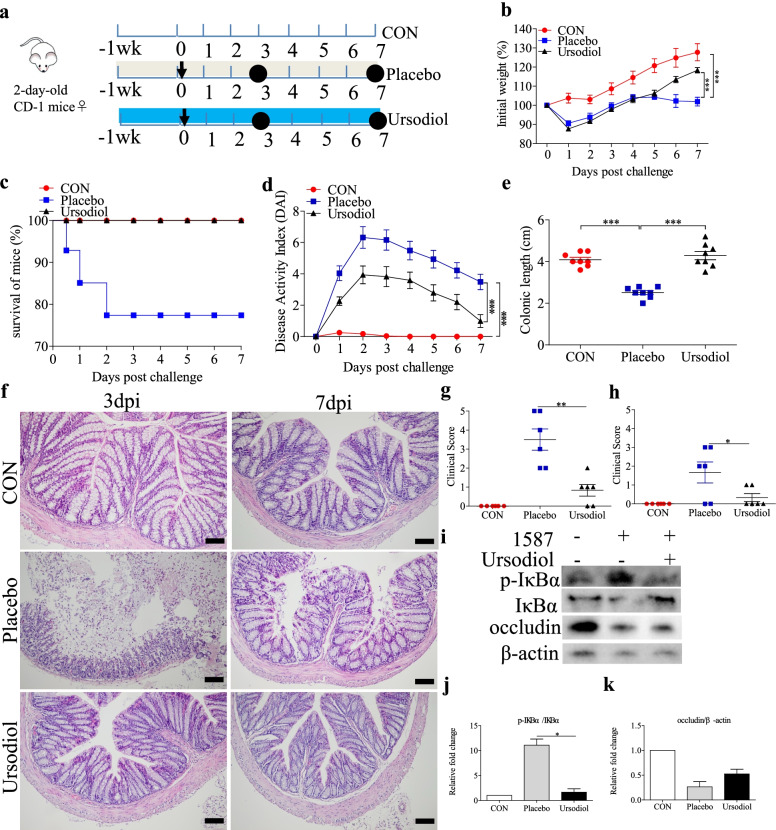
Oral ursodiol alleviated ESBL-EAEC-induced colitis in a neonatal mouse model of peritonitis sepsis. **a** Illustration of the mouse infection model. Oral corn oil and ursodiol before *E. coli* 1587 infection are indicated. Arrows represent the time-point of infection; circles represent the harvest time. **b** Daily body weight changes throughout the entire study. Data are shown as means ± SEM (*n* = 12 per group). Statistical significance was determined using two-way ANOVA. *P ≤ 0.05, **P ≤ 0.01, ***P ≤ 0.001 compared with the placebo group. Survival rate (**c**) and DAI score kinetics (**d**) for the entire study. Survival rate was determined using the log-rank (Mantel-Cox) test. **e** Colonic lengths from each group. Hematoxylin and eosin (H&E)-stained colonic tissues (**f**), histological scores of mouse colons harvested at 3 dpi (**g; ***n* = 6 per group) and 7 dpi (**h; ***n* = 6 per group). **i** Western blot analysis of the NF-κB pathway activation and cell tight junctions. Relative fold changes of p-IκBα (**j**) and occludin (**k**) are presented as means ± SEM. Statistical significance was analyzed using unpaired *t*-tests. *P ≤ 0.05, **P ≤ 0.01, ***P ≤ 0.001

To further study the alleviating effects of ursodiol on ESBL-EAEC infection in vivo, 2-day-old mice were orally gavaged with ursodiol daily for 14 days, then challenged with *E. coli* 1587 via intraperitoneal injection (Fig. 5a). Clinical symptoms in the mice were measured daily using three methods to clarify how ursodiol affected the ESBL-EAEC pathogenic process. Compared with the control and placebo groups, oral ursodiol intervention dramatically alleviated the *E. coli* 1587-induced body weight loss (Fig. 5b), high morbidity (Fig. 5c), and colitis (Fig. 5d) as evidenced by the significantly decreased DAI (comprehensive evaluation score of body weight loss, rectal bleeding, and stool consistency). Adding ursodiol markedly relieved the colonic growth inhibition induced by *E. coli* 1587 infection (Fig. 5e). Histological analysis revealed notable attenuation of the deep mucosal muscularis ulceration, glandular structure destruction, inflammatory cell infiltration (Fig. 5f), and overall colon clinical scores in response to oral gavage with ursodiol at 3 and 7 days post-infection (dpi; Fig. 5g, h).

On the basis of previous reports and our results, intestinal inflammatory reactions were tightly correlated with ESBL-EAEC pathogenesis. Thus, proinflammatory and anti-inflammatory cytokine production levels in the serum and colonic tissues were measured using enzyme-based assays and real-time PCR, respectively. Herein, serum concentrations of IL-1β were markedly suppressed in ursodiol-treated mice after *E. coli* 1587 infection (Additional file 6: Fig. S6a, b), and IL-10 concentrations were prominently upregulated (Additional file 6: Fig. S6c, d). Similarly, *E. coli* 1587 infection stimulated upregulation of IL-1β, IL-6, IL-10, and TNF-α mRNA transcription levels in mouse colons at 3 dpi (Additional file 6: Fig. S6e, g, i, m), but this phenomenon mostly diminished in 7 dpi except for IL-6 (Additional file 6: Fig. S6f, h, j, n). Oral ursodiol administration significantly reduced IL-1β and TNF-α upregulation (Additional file 6: Fig. S6e, m), while IL-10 mRNA expression levels were increased (Additional file 6: Fig. S6j). Besides, *E. coli* 1587 infection induced TGR5 downregulation, and ursodiol exposure significantly increased TGR5 transcription levels (Additional file 6: Fig. S6o). Furthermore, the relative phosphorylation levels of IκBα protein in the colonic tissues were detected using specific antibodies. In similar, oral ursodiol markedly blocked the *E. coli* 1587-induced increases in IκBα phosphorylation (Fig. 5i, j), and ursodiol pretreatment upregulated the occludin protein expression levels (Fig. 5i, k).

SCFA-producing bacteria play vital roles in colonic health in humans, and the main SCFA producers are gram-positive Firmicutes [29]*.* Our previous studies showed that *Butyricicoccus*, *Faecalibacterium*, *Ruminococcus*, *Collinsella*, and *Coriobacterium* were particularly sensitive to ESBL-EAEC. To further investigate how oral ursodiol affects SCFA production, the concentrations of fecal acetate, propionate, and butyrate were detected using a gas chromatography system. *E. coli* 1587 dramatically decreased acetate and butyrate production, but not propionate production. However, only acetate recovered to baseline after oral ursodiol administration at 3 and 7 dpi (Additional file 8: Fig. S8a, c). Neither *E. coli* 1587 nor oral ursodiol affected fecal propionate production (Additional file 8: Fig. S8b). Oral ursodiol strongly suppressed *E. coli* 1587 colonization over the 7-day time period (Additional file 9: Fig. S9a).

Collectively, these results demonstrate that oral ursodiol alleviated the infection symptoms, colitis, and bacterial colonization in the intestines of the neonatal mouse models of peritonitis sepsis. Increased SCFA production in ESBL-EAEC-infected mice in response to oral ursodiol suggested amelioration of the hindgut microbiota disorder and a vital role of the commensal microbiota in relieving ESBL-EAEC infection symptoms.

### Ursodiol administration alleviated colitis after ESBL-EAEC infection in a neonatal mouse oral-infection model

**Fig. 6:**
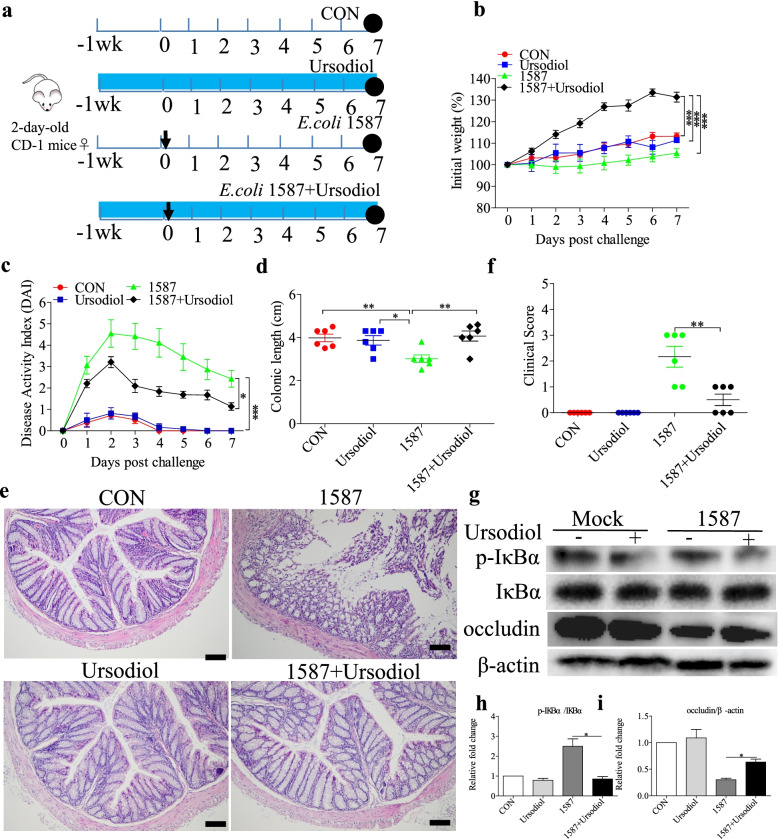
Ursodiol intervention alleviated ESBL-EAEC-induced colitis in a neonatal mouse oral-infection model. **a** Illustration of the mouse infection model. Arrows represent the time-point of infection; circles represent the harvest time. **b** Daily body weight changes post-treatment. Data are presented as means ± SEM (*n* = 6 per group). Statistical significance was determined using two-way ANOVA. *P ≤ 0.05, **P ≤ 0.01, ***P ≤ 0.001 relative to the 1587+ursodiol-treated group. **c** DAI score kinetics for the entire study. **d** Colon lengths from each group. H&E-stained colonic tissues (**e**) and histological scores (**f; ***n* = 6 per group). **g** Western blot analysis of the NF-κB pathway activation and cell tight junctions. Relative fold changes of p-IκBα (**h**) and occludin (**i**) are presented as means ± SEM. Statistical significance was analyzed using unpaired *t*-tests. *P ≤ 0.05, **P ≤ 0.01, ***P ≤ 0.001

The infection routes can alter the virulence of *E. coli* clinical isolates in neonatal septicemia, and oral inoculation is a crucial route for the virulence properties in newborn animals. Oral infection routes can also enhance the ability of *E. coli* to invade and transcytose the intestinal epithelium [36]. We explored how prophylactic ursodiol supplementation via oral delivery affects ESBL-EAEC-related colitis. Two-day-old mice were orally gavaged with ursodiol daily for 14 days, then with *E. coli* 1587 (Fig. 6a). Body weight and disease symptoms were monitored daily, and the colonic pathology was evaluated. Ursodiol intervention mitigated the clinical symptoms in *E. coli* 1587-infected mice as indicated by increased body weight (Fig. 6b), reduced daily DAI (Fig. 6c), and rescued colonic growth and development suppression (Fig. 6d). Histological analysis revealed alleviated inflammatory cell infiltration, colonic edema, mucosal damage (Fig. 6e), and overall colonic clinical scores in response to oral gavage of ursodiol at 7 dpi (Fig. 6f). Expressions of several representative inflammatory cytokines were also detected. Oral ursodiol decreased the serum IL-1β expression levels (Additional file 7: Fig. S7a) and induced a higher concentration of IL-10 (Additional file 7: Fig. S7b). Similarly, *E. coli* 1587 infection elevated the transcription levels of IL-1β (Additional file 7: Fig. S7c), IL-6 (Additional file 7: Fig. S7d), TNF-α (Additional file 7: Fig. S7e), and IL-10 (Additional file 7: Fig. S7f) in the colonic tissues, while TGF-β was decreased (Additional file 7: Fig. S7g). Ursodiol administration recovered only IL-1β and TGF-β expressions to normal levels (Additional file 7: Fig. S7c, g). Besides, *E. coli* 1587 infection downregulated TGR5 expression, and ursodiol exposure significantly increased TGR5 transcription levels (Additional file 7: Fig. S7h). In similar, ursodiol intervention markedly suppressed the increases in IκBα phosphorylation after *E. coli* 1587 infection (Fig. 6g, h), and occludin expression was upregulated (Fig. 6g, i).

As before, we investigated how ursodiol intervention affects SCFA production, especially the concentrations of fecal acetate, propionate, and butyrate. Acetate and butyrate concentrations were decreased in the *E. coli* 1587-induced group relative to those of the control and ursodiol groups, but only acetate was recovered in mice pretreated with ursodiol (Additional file 8: Fig. S8d, f). Conversely, neither oral ESBL-EAEC infection nor ursodiol administration affected propionate production in the colonic contents (Additional file 8: Fig. S8e). Oral UDCA suppressed *E. coli* 1587 colonization in colonic tissues (Additional file 9: Fig. S9b).

Above all, these results indicated that oral UDCA effectively ameliorated infection symptoms, colitis, and bacterial colonization in the hindguts of a neonatal mouse oral-infection model. More specifically, enriched acetate production in ESBL-EAEC-infected mice in response to oral UDCA administration suggested attenuation of the gut microbiota dysbiosis and upregulation of SCFA production in the hindguts of these mice.

### FMT from ursodiol-treated donor mice prevented ESBL-EAEC-induced colitis in neonatal mice

**Fig. 7:**
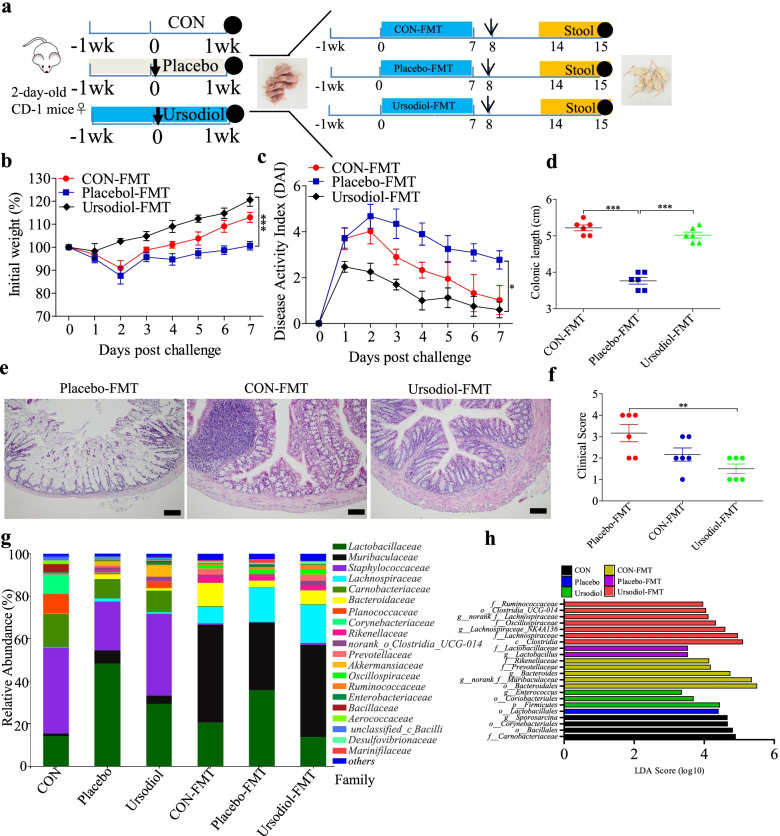
Ursodiol-FMT alleviated ESBL-EAEC-induced colitis better than did the control-FMT and placebo-FMT. **a** Diagram of the neonatal mouse model. Ursodiol-FMT, control-FMT, and placebo-FMT are indicated. Arrows represent the time-point of infection; circles represent the harvest time. **b** Daily body weight changes throughout the entire study period postinfection. Data are presented as means ± SEM (*n* = 6 per group). Statistical significance was determined using two-way ANOVA. *P ≤ 0.05, **P ≤ 0.01, ***P ≤ 0.001 relative to the placebo-FMT group. **c** DAI score kinetics for the entire study. **d** Colon lengths for each group. H&E-stained colonic tissues (**e**) and histological scores (**f; ***n* = 6 per group). **g** Relative abundances of fecal bacterial families in 99.5% of the community. **h** Analysis of differences in enriched microbiota taxa shown by LEfSe (linear discriminant analysis (LDA) coupled with effect size measurements) upon FMT

Initial reports claimed that reconstitution of a healthy gut microbial community is closely related to normal gastrointestinal status [37, 38]. Our previous results suggested that the gut microbiota is vital in relieving ESBL-EAEC infection symptoms. Thus, we examined how the ursodiol-treated microbiota affects *E. coli* 1587-induced colitis by transplanting the fecal microbiotas from neonatal mice undergoing ursodiol oral gavage in response to *E. coli* 1587 infection (Fig. 7a). Here, fresh feces from the control, placebo and ursodiol-treated groups were collected and transplanted in neonatal mice. The body weight and DAI were monitored daily from days 8 to 14, and fresh feces were collected to analyze the gut microbiota profiles. Similarly, ursodiol-FMT significantly attenuated the clinical symptoms in *E. coli* 1587-infected mice as indicated by significantly increased body weight (Fig. 7b), decreased daily DAI (Fig. 7c), and recovery of normal colonic growth and development (Fig. 7d). Histological analysis further revealed dramatic alleviation of inflammatory cell infiltration, colonic edema, mucosal damage (Fig. 7e), and overall colonic clinical scores in response to ursodiol FMT compared with those of the placebo and control groups (Fig. 7f).

We detected the fecal microbiota compositions post-FMT using 16S rRNA gene analysis. Chao1 and Shannon indexes of the α-diversity between groups showed that FMT significantly increased the bacterial community richness compared with that of the corresponding donor mice, and the α-diversity of the ursodiol-FMT-treated mice increased compared with that of the placebo-FMT-treated group (Additional file 10: Fig. S10a, b). Microbial compositions differed between groups at the phylum and family levels, including reduced Firmicutes, Actinobacteria, and Verrucomicrobiota and increased Bacteroidetes in the FMT-treated groups. Enterobacteriaceae was increased in both the placebo and placebo-FMT groups (Additional file 10: Fig. S10c, Fig. 7g). Besides, Lactobacillaceae was decreased, and Bacteroidaceae and Muribaculaceae were enriched in the ursodiol-FMT group compared with those of the placebo-FMT group. PCoA based on weighted UniFrac distance indicated dispersed data points on the plots of the donor groups (*R*^2^ = 0.6492, *P* = 0.001) and among recipient groups (*R*^2^ = 0.3345, *P* = 0.008; Additional file 10: Fig. S10d, e). LEfSe analysis showed that oral ursodiol increased the abundances of Firmicutes, Coriobacteriales, and *Enterococcus*. Meanwhile, Ruminococcaceae, Oscillospiraceae, Lachnospiraceae, and *Clostridia_UCG-014* were significantly enriched after ursodiol-FMT, and *Lactobacillus* was enriched in the placebo and placebo-FMT groups. This result was similar to the microbial profiles for female neonatal calves (Fig. 7h). Next, we investigated the fecal acetate, propionate, and butyrate productions in mouse donors and recipients. ESBL-EAEC infection markedly reduced the acetate and butyrate productions; only acetate was recovered after oral ursodiol, and propionate was nearly unchanged (Additional file 11: Fig. S11a–c). Interestingly, ursodiol-FMT markedly upregulated the acetate and propionate productions, but not butyrate production, compared with those of the placebo-FMT group (Additional file 11: Fig. S11d–f).

Spearman correlation analysis was performed to interpret the relevance between differentially enriched bacteria and SCFA production. *Unclassified__Clostridia_UCG-014* was strongly positively correlated with acetic acid (*R* > 0.55, *P* = 0.018) and propionic acid (*R* > 0.53, *P* = 0.021), thus validating the LEfSe results of the FMT and revealing the beneficial effect of ursodiol on commensal microbial SCFA production. *Muribaculum* was strongly positively correlated with acetic acid (*R* > 0.54, *P* = 0.020) and propionic acid (*R* > 0.50, *P* = 0.033), and *Candidatus saccharimonas* was correlated with enriched butyric acid (*R* > 0.71, *P* = 0.00097). Acetic acid abundance was correlated with *Parabacteroides* (*R* > 0.52, *P* = 0.025) and *Bacteroides* (*R* > 0.56, *P* = 0.014; Additional file 12: Fig. S12). *Lactobacillus* was strongly negatively correlated with acetic acid (*R* < − 0.62, *P* = 0.0060) and propionic acid (*R* < − 0.61, *P* = 0.0065), which may increase lactic acid levels. This result was similar to the abundant lactic acid and *Lactobacillus* in diarrheic calves in our previous study (Figs. 1b and 2b).

Overall, ursodiol-FMT directly ameliorated clinical symptoms and colitis in ESBL-EAEC-induced neonatal mice. Ursodiol-FMT simultaneously induced the beneficial effects of enriched Ruminococcaceae, Oscillospiraceae, Lachnospiraceae, and *Clostridia_UCG-014* and increased SCFA production, indicating the direct alleviating effects of an improved commensal microbiota structure in ursodiol-treated mice on clinical symptoms and colitis as well as direct antibacterial effects against ESBL-EAEC invasion.

## Discussion

DEC is frequently isolated from sporadic clinical diarrhea cases and in gastroenteritis outbreaks worldwide [39]. DEC can be categorized into six pathotypes: enteropathogenic *E. coli* (EPEC), enterohaemorrhagic (Shiga-toxin producing) *E. coli* (EHEC/STEC), enterotoxigenic *E. coli* (ETEC), enteroaggregative *E. coli* (EAEC), enteroinvasive *E. coli* (EIEC) and diffusely adherent *E. coli* (DAEC) [40]. Among these, the pathogenesis of ESBL-EAEC remains unclear, and excessive antibiotic misuse accelerates the rapid spread of multidrug-resistant bacteria. In fact, multidrug-resistant DEC causes extensive diarrheal infections among nonhuman animal and human hosts, especially owing to the increasing occurrence of ESBL-producing strains [41]. Herein, to help alleviate suffering from ESBL-EAEC infections in neonatal dairy calves, we investigated UCDA as an antibiotic alternative to prevent further resistance dissemination. Thus, clinical ESBL-EAEC isolate was isolated from diarrheic neonatal dairy calves in a conventional pasture, who harbored various antimicrobial genes and enterotoxin EAST1, which possesses cell adhesion ability. In fact, EAST1-expressing strains have been shown to induce diarrhea primarily in humans, and then rapidly adapt and propagate in piglets, calves, and other animals [42]. In most cases, ESBL-EAEC infections are self-limiting and asymptomatic in the host, but some environmental factors, such as colostrum, genetics, and gut microbiota diversity, correlate closely with development and progression of calf diarrhea [1]. Currently, we separated only EAEC strains owing to geographic restrictions and limited large ranch opening permissions. Therefore, our data revealed only the antibacterial effects of UDCA on ESBL-EAEC. In our subsequent clinical research, we will investigate the effects of UDCA on the pathogenesis of other DEC strains and isolate these stains from calves on other conventional pastures in the other parts of China. Future studies will systematically clarify whether UDCA is a superior antibiotic alternative for early intervention in improving young ruminant healthcare with reduced DEC transmission.

Importantly, dietary preferences greatly affect the changes in gut microbial diversity in healthy dairy calves [43]. The calves in our study were fed only milk replacer (same intake at the same age), and neonatal mice were fed breast milk from littermate female mice to detect the specific host biomarkers that continuously influenced ESBL-EAEC occurrence and progression in their early lives. To accurately understand how ESBL-EAEC affects the fecal microbiome (taxonomy and structure), we compared the bacterial compositions among the H_1, H_2, D_1, and D_2 groups, considering that most diarrhea cases occurred in newborn calves from 4 to 11 days of age during our 3-week study. Relative abundances of the total bacterial genera did not differ, and the Shannon and Chao1 indexes were similar in the H_1 vs H_2 and D_1 vs D_2 phases. However, gut microbiome structures were greatly altered. The commensal microbiota acts as a ligament between endogenous and exogenous factors [44] and contributes to nutrient acquisition, pathogen exclusion, and immune recognition [45]. Here, the findings of this study indicated that ESBL-EAEC promoted the diarrhetic process by altering the hindgut commensal compositions, including severely reducing the abundances of Coriobacteriaceae, Ruminococcaceae, Lachnospiraceae, further decreasing UDCA levels and destroying the bile acid pool in the hindgut digesta. These findings are similar to the changes in the gut microbiotas of humans with and without inflammatory bowel diseases [46]. The D_1 and D_2 groups contained higher relative abundances of *Streptococcus* and *Flavobacterium*, respectively. Indeed, higher *Streptococcus* prevalence has been reported to correlate with quantitative diarrhea outcomes except in DEC infections [47]. Besides, *Flavobacterium* is a common species among bacterial communities in diarrhea-affected cattle [48]. In the first phase (1–7 days), Enterobacteriaceae/*E. coli-Shigella* were not enriched in the D_1 group compared with those of H_1. However, in the second phase (7–14 days), Enterobacteriaceae/*E. coli-Shigella* were largely abundant in the D_2 group compared with those of the H_2 group. Several reasons may explain the gradual growth of Enterobacteriaceae/*E. coli-Shigella* over time. First, the existing maternal antibodies still protected neonatal calves from pathogenic Enterobacteriaceae/*E. coli-Shigella* colonization in the first phase. Furthermore, the ESBL-EAEC was just starting to colonize in the intestinal epithelium and release small numbers of virulence factors, facing competitive inhibition from commensal bacteria. Over time, increasing replicative ESBL-EAEC colonized and became dominant over the commensal bacteria, thus releasing abundant diarrhea-associated virulence factors. While, in the healthy groups, the gradually enriched UDCA and other antibacterial metabolites significantly reduced the relative abundances of Enterobacteriaceae*/E. coli-Shigella* over time and inhibited pathogenic ESBL-EAEC.

The hindgut microbial population was characterized by the dominance of lactic acid bacteria, including *Bifidobacterium*, *Lactobacillus*, and *Streptococcus*, as indicated by enriched lactic acid production in the diarrheic groups. Similar to previous findings [49], neonatal Holstein calf hindgut fermentation could take prominent position in metabolizable energy supply during the first weeks of life in comparison with ruminal fermentation, because of the active reticular groove. Actually, decreased commensal bacteria such as *Butyricicoccus*, *Faecalibacterium*, *Ruminococcus*, *Collinsella*, and *Coriobacterium* correlated strongly with the reduced UDCA. Importantly, our results revealed a higher prevalence of SCFA-producing bacteria belonging to Ruminococcaceae (containing *Faecalibacterium* and *Butyricicoccus*) and Lachnospiraceae in healthy calves [32, 50–53], with upregulated butyric acid levels in the H_1 group compared with those of the D_1 group. Thus, the beneficial effects of butyric acid on the gut mucosal immune responses provided adjunctive effects against ESBL-EAEC infection [54, 55]. Future studies should use culturing techniques, proteomics, and bile acid-targeted metabonomics to clarify how these hindgut commensal bacteria mediate high UDCA expressions, directly alleviate colitis and confer antibacterial effects in ESBL-EAEC-induced diarrheic neonatal calves after direct administration of bacteria purely isolated from healthy calves.

Bile acids, specific natural cholesterol-derived amphipathic molecules of saturated hydroxylated C-24 sterols, are synthesized primarily by hepatocytes [56]. Biotransformation of bile acid is a collaborative effort between the host and gut microbiota. The gut microbiota modifies host-derived primary bile acids into secondary bile acids once they enter the gastrointestinal tract. In fact, nearly all bile acids are microbially derived in the colon, and various bacteria can dehydrogenate unconjugated bile acids [57]. The primary bile acids in humans are cholic acid (CA) and chenodeoxycholic acid (CDCA) in human. Secondary bile acids, such as lithocholate (LCA) and deoxycholate (DCA), can be cytotoxic molecules inducing colonic carcinogenesis, membrane damage, and oxidative stress [58].

UDCA is pleasingly found to protect colonic epithelial cells from oxidative damage and cell apoptosis [58]. The FDA-approved commercial formulation of UDCA, ursodiol, is mostly used to treat primary biliary cirrhosis, cholangitis, nonalcoholic fatty liver disease, cholesterol gallstones, primary sclerosing cirrhosis [11, 13, 59–61], and for *Clostridioides difficile infection* (CDI) patients curation [15, 62]. However, limited evidence supports the direct use of ursodiol to treat ESBL-EAEC infections. Here, ursodiol administration dose-dependently inhibited ESBL-EAEC growth, reduced the adherence of this strain in vitro and significantly decreased intestinal bacterial colonization in vivo. This suggests that ursodiol can directly block ESBL-EAEC invasion, and exogenous administration of ursodiol may confer colonization resistance against ESBL-EAEC infection in vivo. Further, we found that fecal concentrations of UDCA were dramatically enriched in the H_1 and H_2 groups compared with those of D_1 and D_2, indicating its continuous effect on ESBL-EAEC occurrence and progression. High-fold increases of other bile acids, such as βUDCA, UCA, and βMCA, were also detected in the healthy groups, suggesting that these bile acids might also protect against infection and modulate bile acid pools to restore colonization resistance against ESBL-EAEC. These findings are similar to the significant modifications in the bile acid metabolome and upregulation of several key bile acid species, such as UDCA, TUDCA, and TβMCA, following ursodiol pretreatment [15]. Apart from the conspicuous upregulations of bile acids and butyric acid, previous results revealed that various benzenoids and indoles, such as phenylacetic acid (PAA), phenylpropionic acid (PPA), gallic acid, indole-3-carboxylic acid, and indole-3-propionic acid, were enriched in healthy groups. In fact, PAA, PPA, and indoles are commonly detected phenolic metabolites in animal feces. PAA and indoles are naturally occurring plant auxins that possess substantial antimicrobial and anti-inflammatory activities [63, 64]. Gallic acid and its derivatives possess anti-inflammatory, antioxidant, and antimicrobial properties [65]. Indeed, except for bile acid and SCFA metabolism, these metabolites may also have probiotic effects during ESBL-EAEC infection. Further research is needed to investigate the mechanisms of action of these metabolites against enteric pathogens in young ruminants. Studies of the effects of bile acid metabolism on gut disorders in livestock are also urgently needed. Compared with those probiotic factors, what was worth taking seriously specially is the prominently abundant hippuric acid in both D_1 and D_2 groups. As a protein-bound uremic toxin [66], accumulated hippuric acid has been closely correlated with stimulation of proinflammatory cytokines and oxidative stress, which can accelerate disease deterioration and indicate its utility in calf fece as a plausible hallmark of frailty post ESBL-EAEC infection.

To explore how UDCA attenuates clinical symptoms and colitis, ursodiol was directly added to Caco-2 cell cultures. Ursodiol exposure directly blocked the growth and cell adherence of *E. coli* 1587 relative to that of the K12 strain and suppressed the transcription levels of IL-6 and TNF-α, with upregulation of IL-10 expression after stimulation with ESBL-EAEC-LPS. In addition, ursodiol addition also protected cell integrity and barrier function from LPS-induced cell damage. Similar to previous findings [15], oral administration of ursodiol ameliorated the clinical symptoms, as demonstrated by reductions in mortality, DAI, body weight loss, and histological damage. Importantly, oral ursodiol also alleviated colonic inflammation by downregulating proinflammatory cytokines IL-1β and TNF-α and upregulating IL-10 and TGF-β expressions in the plasma and colonic tissues.

As is known to all, the NF-κB signaling pathway is a prominent innate immune pathway activated by LPS in intestinal epithelial cells and induces transcription of IL-1β, IL-6, and TNF-α [67]. Pretreatment with ursodiol suppressed IκBα phosphorylation and enhanced tight junctions. These findings were not surprising considering the fact that bile acid-related receptors adjust host innate immune responses [68]. Bile acids and gut microbiota interactions alter bile acid pools, thus regulating signaling via the FXR and TGR5 receptors, which can modulate innate immune responses by inhibiting NF-κB elements [68–70]. We detected TGR5 gene transcription levels in Caco-2 cells and neonatal mouse infection models and found that ESBL-EAEC infection downregulated TGR5, and ursodiol exposure significantly increased TGR5 transcription levels, thus mediating further inhibition of IκBα phosphorylation. More importantly, bile acid concurrently activates both pathways, reduces transcription of IL-1β, IL-6, and TNF-α via the NF-κB signaling pathway [71], and regulates IL-10 expression levels [72]. These findings indicate that the anti-gastric inflammatory effect of UDCA mainly relies on TGR5 activation and the subsequent inhibition of NF-κB signaling after ESBL-EAEC infection.

Previous publications reported major age-dependent microbial and metabolic changes and identified bile acids as potent drivers of early intestinal microbiota maturation, thus indicating the potential of bile acids to regulate gut microbiota structure [73]. This is similar to our finding that UDCA was highly correlated with enriched probiotics and fewer *E. coli-Shigella* (especially in the H_2 phase), *Streptococcus* and *Lactobacillus* (producing abundant lactic acid). Different animals have highly diverse digestive systems, gut microbiomes, and numbers and profiles of immune cells depending on development or aging processes. Here, we detected the anti-ESBL-EAEC effects and colitis-alleviating abilities of UDCA in neonatal mice. The study had some shortcomings, especially in the direct effect of UDCA on young ruminant T cell maturation of the lamina propria and gut microbial maturation in neonatal ruminants. We will examine these problems in future studies using neonatal calf UDCA feeding trials. Systematically understanding the exact molecular mechanisms of UDCA in neonatal calf physiology, including colitis attenuation, attenuation of hindgut bacterial damage, and SCFA production in the digesta after ESBL-EAEC infection, as well as T cell maturation of the lamina propria and long-term effects on gut microbe maturation in neonatal ruminants, will help accelerate UDCA applications in the livestock industry.

Importantly, Whon et al. highlighted that gut inflammation followed by a prolonged expansion of autochthonous Enterobacteriaceae contributed to the onset of calf diarrhea [74]. This was similar to our results for Enterobacteriaceae replication over time under ESBL-EAEC exposure. Besides, these authors also showed the emergence of inflammation-resistant or antibiotic-resistant microbes via active horizontal gene transfer mediated by lytic bacteriophages. Furthermore, clinical ESBL-producing plasmids spread in the absence of antibiotics and in the complex intestinal environment in animals and humans, especially between *E. coli* and *E.coli* and between *E. coli* and *Salmonella* [75]*.* In our previous study, we detected obvious plasmid-mediated ESBL-gene transfer, which mediated antibiotic resistance in recipient cells. Fortunately, the isolated ESBL-EAEC strain 1587 also induced bacterial infection and colitis in neonatal mice despite species barriers, thus suggesting that bovine ECBL-EAEC could also spread among different mammals mainly through plasmid-mediated antibiotic-resistant gene transfer. Here, the positive rate of ESBL-EAEC was very high among neonatal dairy calves and induced high mortality and morbidity in local conventional pasture. This is likely to occur in other pastures in other parts of China. Hence, timely control and prevention of ESBL-EAEC among calves are urgently needed.

Nowadays, FMT is a highly effective therapy for treating *Clostridioides difficile* infection (CDI), inflammatory bowel disease (IBD), and irritable bowel syndrome (IBS) [76, 77] and has been applied in both monogastric animals [78–80] and ruminants [81]. Thus, we used FMT to investigate whether oral ursodiol affected the gut microbiota compositions and their derived metabolites in ESBL-EAEC-infected mice. We explored the beneficial effects of ursodiol-FMT in neonatal mice. Interestingly, ursodiol-FMT directly ameliorated clinical symptoms and colitis, which was consistent with the effects of oral ursodiol. Importantly, it showed better alleviation of symptoms than did the control-FMT, suggesting that ursodiol-mediated hindgut microbiome alterations are vital during ESBL-EAEC infections. Here, FMT had significant accelerative effects on bacterial community richness. Ursodiol-FMT greatly influenced the microbial structure compared with that of the donors, which might be attributed to its prebiotic characteristics. Interestingly, it dramatically upregulated Oscillospiraceae, Ruminococcaceae, Lachnospiraceae, and *Clostridia_UCG-014* and promoted SCFA production. These results suggest that ursodiol-FMT had a similar effect to that of ursodiol itself, indicating that an improved microbiota was conducive to treating ESBL-EAEC-induced colitis. FMT therapy shows great potential for treating most colibacillus diarrhea. Based on above empirical results, we concluded that the immediate block of ESBL-EAEC growth and invasion, alterations of the gut microbiota, and SCFA production mediated by ursodiol intervention were the key reasons for the alleviation of ESBL-EAEC infection. Bile acid pool modulation has been used as a rational therapeutic option in restoring colonization resistance against CDI by using traditional approaches such as FMT [82] or key commensal supplementation [83]. In the current study, our results indicate that direct intervention with specific bile acids, such as ursodiol, may help restore or partially restore colonization resistance against some enteric pathogens and may modulate host inflammatory responses.

In conclusion, ESBL-EAEC infection induced temporal alterations in the hindgut microbial community and fecal metabolites in neonatal dairy calves. The negative effects of ESBL-EAEC infection on animal health were correlated with gut microbiota damage and prominently reduced levels of the microbiota-derived metabolite, UDCA, destruction of bile acid pools, and reduced SCFAs and other critical prebiotics. UDCA was evidenced to block bacterial growth and invasion processes, mediating intestinal homeostasis by improving hindgut microflora structure and SCFA production during ESBL-EAEC infection in sepsis and colitis in neonatal mouse models. Our findings not only revealed the potential hazards and risks of ESBL-EAEC infection in these young animals, but also suggested UDCA as a potential antibiotic alternative for future intervention of ESBL-EAEC infections.

## Conclusion

Multi-omics analyses of fecal samples from growing neonatal calves revealed differences in the gut microbiota and associated metabolites. ESBL-EAEC-induced diarrhea in neonatal calves influenced the gut microbiota structure, particularly for the commensal bacteria *Butyricicoccus*, *Faecalibacterium*, *Ruminococcus*, *Collinsella*, and *Coriobacterium* and was accompanied by changes in UDCA metabolism and bile acid pools and decreased production of SCFAs and other prebiotics. ESBL-EAEC infection induced TGR5 downregulation, and ursodiol exposure significantly increased TGR5 transcription levels, thus further inhibiting IκBα phosphorylation. Importantly, ursodiol administration inhibited ESBL-EAEC growth and blocked cell adherence. Both oral ursodiol and FMT significantly ameliorated gut microbiota damage, clinical symptoms, and colitis and induced blockage of enteric pathogenic colonization in the colons of neonatal mice. These changes were likely achieved by reconstruction of colonization resistance, mainly through aggregated Oscillospiraceae, Ruminococcaceae, Lachnospiraceae, and *Clostridia_UCG-014* and upregulation of SCFA production. These findings provided innovative insight into the diverse antibacterial effects of UDCA during ESBL-EAEC infection and could probably act as powerful tool facilitating the reduction of ESBL-EAEC transmission and antimicrobial usage in the livestock industry.

## Supplementary Information

**Additional file 1: Fig. S1.** Effect of ESBL-EAEC infection-driven gut microbiota assembly in diarrheic calves. Relative abundance of fecal bacterial phyla (a) and families (b) in 99.5% of the community. Enriched gut microbiota taxa are shown by LEfSe (linear discriminant analysis (LDA) coupled with effect size measurements) of H_1 vs D_1 (c) and H_2 vs D_2 (d).

**Additional file 2: Fig. S2.** KEGG analysis of differentially expressed genes of H_1vs D_1 (a) and H_2 vs D_2 (b). The name of each KEGG pathway is shown on the left; pathway categories are indicated on the right.

**Additional file 3: Fig. S3.** Fecal metabolomic analysis revealed metabolite alterations in diarrheic calves. (a) Total metabolome classifications of compounds with differential metabolites in the H_1, H_2, D_1 and D_2 groups. The total number of significantly changed metabolites in this class are indicated, and the corresponding proportions are in parentheses. (b) Fecal metabolome profiles of calves were clustered using three-dimensional PLS-DA. Metabolomic profiles for the H_1, H_2, D_1 and D_2 groups are shown in the same colors. Fecal metabolomic profiles of calves were clustered using PCA with boxplot (c) and three-dimensional PCA (d). The metabolomic profiles for the H_1, H_2, D_1 and D_2 groups are shown in the same colors. Data are presented as the mean±SEM. *P*-values were determined using the nonparametric Kruskal-Wallis test. (e) KEGG pathway enrichment analysis according to the markedly altered metabolites. The name of each KEGG pathway is shown on the left; the corresponding *P*-value is shown on the right and represented by a gradient color. *P-*values were determined using two-sided Fisher’s exact tests with Benjamini-Hochberg correction for multiple testing. (f) Random forest supervised machine-learning algorithm of metabolites in the H_1, H_2, D_1, and D_2 groups. The name of each metabolite is shown on the left. Top 10 metabolites in the fecal samples are shown in different colors; rank values are presented as MeanDecreaseGini.

**Additional file 4: Fig. S4.** Metabolomic analyses of fecal samples from healthy and diarrheic calves. Partial least squares discriminant analysis (PLS-DA) for dairy calves in H_1 vs D_1 (a), H_2 vs D_2 (b), H_1 vs H_2 (c), D_1 vs D_2 (d). H, healthy calves; D, diarrheic calves.

**Additional file 5: Fig. S5.** Volcano maps of fecal metabolomics from healthy and diarrheic calves. Enriched metabolites were identified by analyses with *P*-values <0.05 and fold change (FC) values ≥1 (| log2FC|≥0). Significantly upregulated and downregulated metabolites are in red and blue, respectively. Metabolites with no obvious changes (noise) are gray. The differentiated metabolites in H_1 vs D_1 (a), H_2 vs D_2 (b), H_1 vs H_2 (c), D_1 vs D_2 (d) are shown. The numbers of upregulated and downregulated metabolites are on the right.

**Additional file 6: Fig. S6.** Expression levels of inflammatory cytokines and TGR5 in a neonatal mouse model of peritonitis sepsis. IL-1β (a, b) and IL-10 (c, d) concentrations in the serum. Relative mRNA expression levels of five representative inflammatory cytokines, IL-1β (e, f), IL-6 (g, h), IL-10 (i, j), TGF-β (k, l), TNF-α (m, n), and TGR5 (o) in the colon. Data are presented as means±SEM. Statistical significance was analyzed using unpaired t-tests. *P≤0.05, **P≤0.01, ***P≤0.001.

**Additional file 7: Fig. S7.** Expression levels of inflammatory cytokines and TGR5 in neonatal mouse oral-infection model. IL-1β (a) and IL-10 (b) concentrations in the serum. Relative mRNA expression levels of five representative inflammatory cytokines, IL-1β (c), IL-6 (d), TNF-α (e), IL-10 (f), TGF-β (g), and TGR5 (h), in the colon. Data are presented as means±SEM. Statistical significance was analyzed using unpaired t-tests. *P≤0.05, **P≤0.01, ***P≤0.001.

**Additional file 8: Fig. S8.** SCFA concentrations in colonic contents after oral therapy. Concentrations of acetate (a, d), propionate (b, e), and butyrate (c, f) upon oral therapy (*n* = 4–6 per group). Data are presented as means±SEM. Statistical significance was analyzed using unpaired t-tests. *P≤0.05, **P≤0.01, ***P≤0.001.

**Additional file 9: Fig. S9.** Colonizations blocks of strain 1587 in colonic tissues of neonatal mouse infection models. Suppressive effect of oral ursodiol on 1587 strain colonization in neonatal mouse sepsis (a) and oral infection (b) models. Data are presented as means±SEM. Statistical significance was analyzed using unpaired t-tests. *P≤0.05, **P≤0.01, ***P≤0.001.

**Additional file 10: Fig. S10.** Gut microbiota profiles of mouse donors and recipients. Alpha-diversity of different groups by Chao1 (a) or Shannon (b) index. Data are presented as the mean±SEM by unpaired t-test. *P≤0.05, **P≤0.01, ***P≤0.001. (c) Relative abundances of fecal bacterial phyla in 99.5% of the community. PCA plots based on the weighted UniFrac distance matrix of FMT donors (d; *n* = 6) and recipients (e; *n* = 6). Data were assessed using PERMANOVA analysis, with 999 permutations.

**Additional file 11: Fig. S11.** SCFA concentrations in colonic contents of neonatal mouse donors and recipients. Concentrations of acetate (a), propionate (b), and butyrate (c) in donors upon oral therapy (*n* = 4 per group). Concentrations of acetate (d), propionate (e), and butyrate (f) in recipients upon FMT (*n* = 6 per group). Data are presented as means±SEM. Statistical significance was analyzed using unpaired t-tests. *P≤0.05, **P≤0.01, ***P≤0.001.

**Additional file 12: Fig. S12.** Spearman correlation between the fecal microbiotas of FMT recipients and SCFA production of acetic, propionic, and butyric acid. Red denotes a positive correlation; blue denotes a negative correlation. Color intensity is proportional to the strength of the Spearman correlation. *P ≤ 0.05, **P≤0.01, ***P≤0.001.

**Additional file 13: Table S1.** Genomic analysis of DEC bacterial strain.

**Additional file 14: Table S2.** Antibiotic susceptibility analysis of *E. coli* 1587 strain.

**Additional file 15: Table S3.** Sampling information of calves used in the current study.

**Additional file 16: Table S4.** Fecal metabolites of dairy calves.

**Additional file 17: Table S5.** Fecal metabolites rank of random forest in dairy calves.

## Data Availability

Metabolomics raw data have been deposited in Mendeley Data (https://data.mendeley.com//datasets/gspw8w9wt6/1). 16S rRNA gene data has been deposited in the National Center for Biotechnology Information (NCBI) Sequence Read Archive (SRA) database under accession number PRJNA767340 and PRJNA780326. Source data are provided with this paper.

## Abbreviations

EAEC: Enteroaggregative *E. coli*
DEC: Diarrheagenic *Escherichia coli*
MDR: Multidrug resistance
ESBL: Extended-spectrum beta lactamase
UDCA: Ursodeoxycholic acid
CA: Cholic acid
CDCA: Chenodeoxycholic acid
LCA: Lithocholate
DCA: Deoxycholate
DSS: Dextran sulfate sodium
CDI: *Clostridioides difficile* infection
LPS: Lipopolysaccharide
SCFA: Short-chain fatty acid
H&E: Hematoxylin and eosin
FMT: Fecal microbiota transplantation
SPF: Specific pathogen-free
DAI: Disease activity index
KEGG: Kyoto Encyclopedia of Genes and Genomes
PCoA: Principal co-ordinates analysis
LDA: Linear discriminant analysis
PAA: Phenylacetic acid
PPA: Phenylpropionic acid
IBD: Inflammatory bowel disease
IBS: Irritable bowel syndrome
