# Supplementary figures and images for: Gut microbiota-derived ursodeoxycholic acid from neonatal dairy calves improves intestinal homeostasis and colitis to attenuate extended-spectrum β-lactamase-producing enteroaggregative Escherichia coli infection
Source: Microbiome. 2022 May 28;10:79. doi: 10.1186/s40168-022-01269-0 (PMC9142728; doi:10.1186/s40168-022-01269-0)

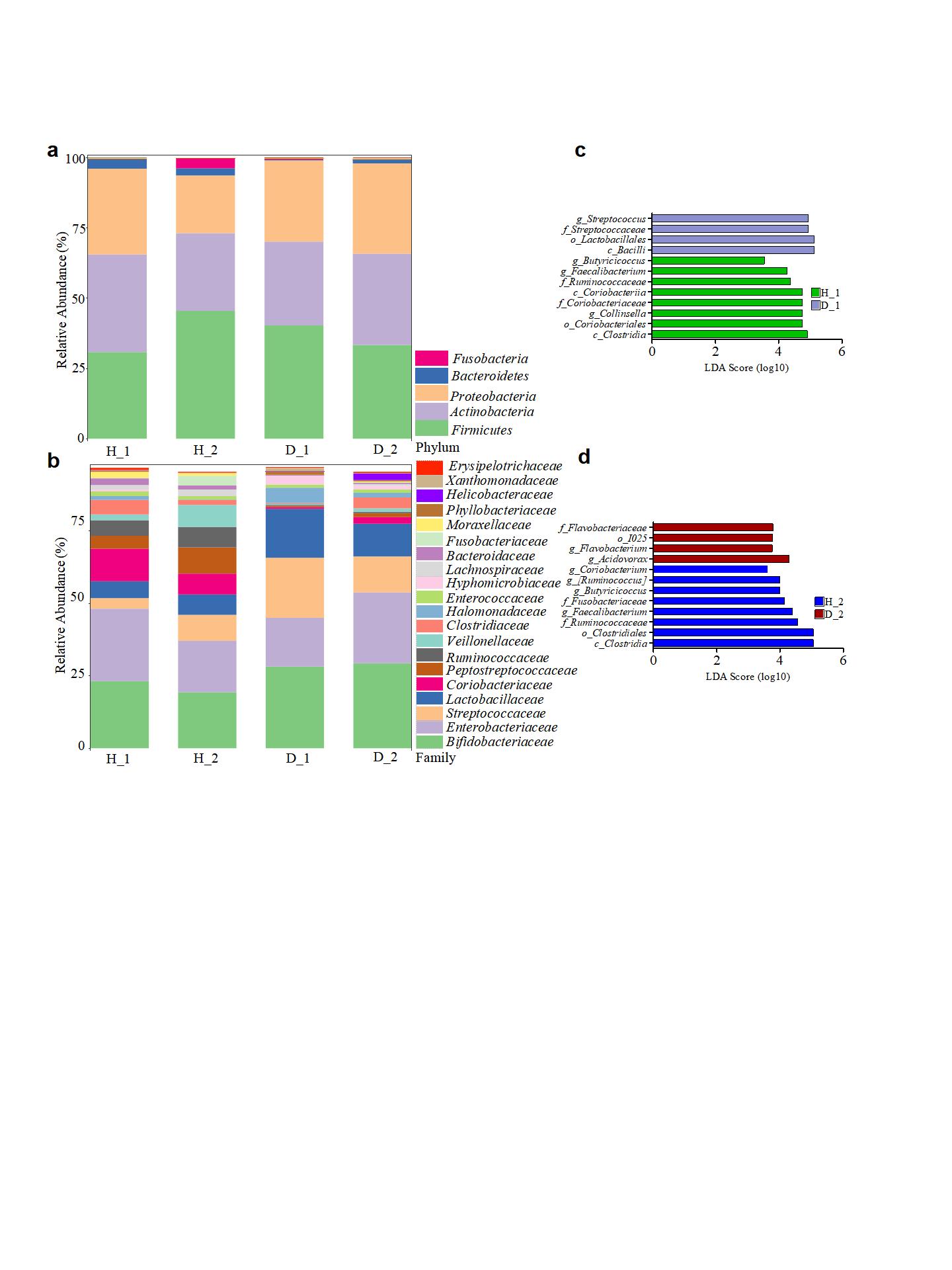

Supplement: Supplementary file 2 — Additional file 1: Fig. S1. Effect of ESBL-EAEC infection-driven gut microbiota assembly in diarrheic calves. Relative abundance of fecal bacterial phyla (a) and families (b) in 99.5% of the community. Enriched gut microbiota taxa are shown by LEfSe (linear discriminant analysis (LDA) coupled with effect size measurements) of H_1 vs D_1 (c) and H_2 vs D_2 (d). [file 40168_2022_1269_MOESM2_ESM.jpg]

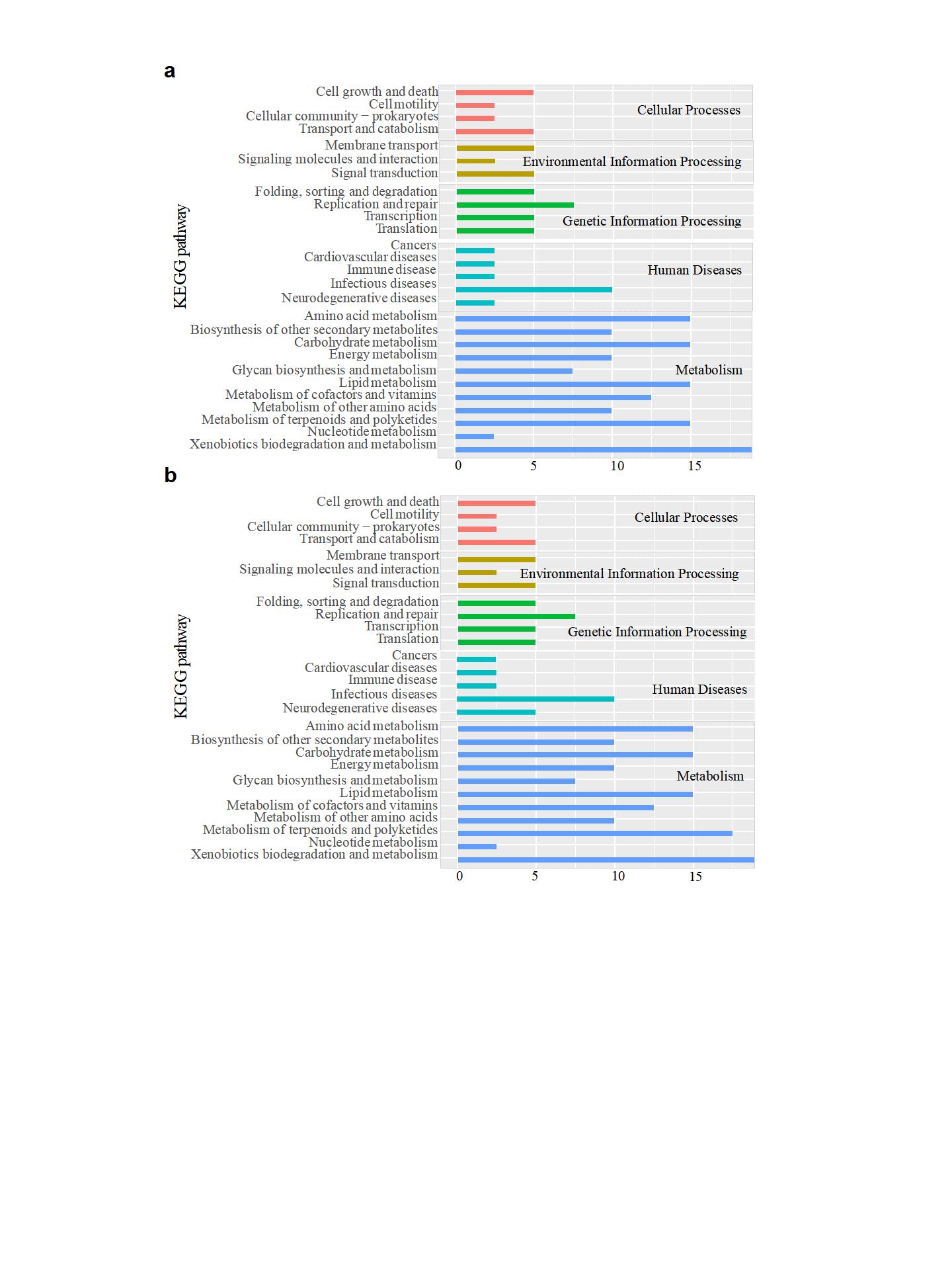

Supplement: Supplementary file 3 — Additional file 2: Fig. S2. KEGG analysis of differentially expressed genes of H_1vs D_1 (a) and H_2 vs D_2 (b). The name of each KEGG pathway is shown on the left; pathway categories are indicated on the right. [file 40168_2022_1269_MOESM3_ESM.jpg]

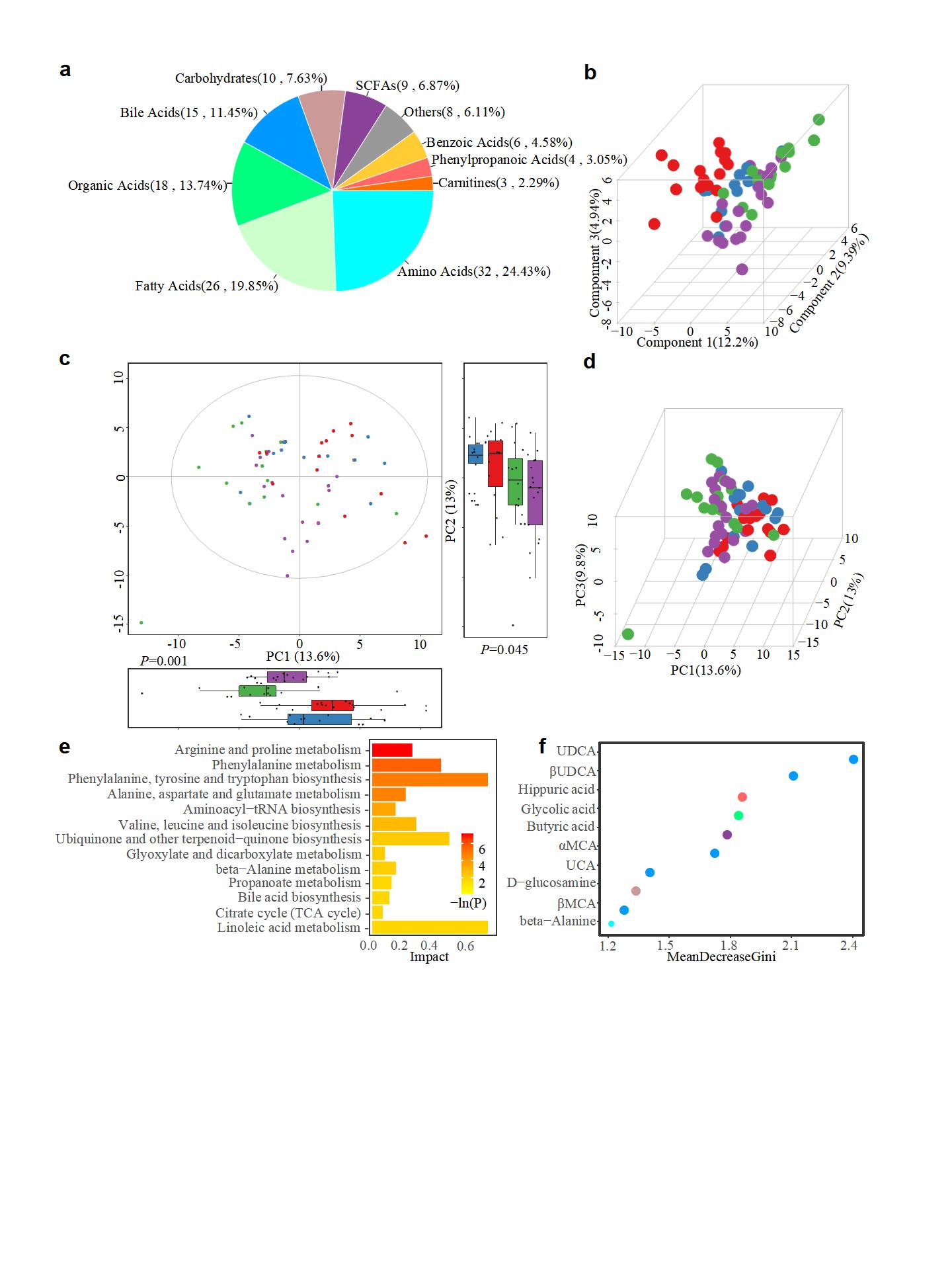

Supplement: Supplementary file 4 — Additional file 3: Fig. S3. Fecal metabolomic analysis revealed metabolite alterations in diarrheic calves. (a) Total metabolome classifications of compounds with differential metabolites in the H_1, H_2, D_1 and D_2 groups. The total number of significantly changed metabolites in this class are indicated, and the corresponding proportions are in parentheses. (b) Fecal metabolome profiles of calves were clustered using three-dimensional PLS-DA. Metabolomic profiles for the H_1, H_2, D_1 and D_2 groups are shown in the same colors. Fecal metabolomic profiles of calves were clustered using PCA with boxplot (c) and three-dimensional PCA (d). The metabolomic profiles for the H_1, H_2, D_1 and D_2 groups are shown in the same colors. Data are presented as the mean±SEM. P-values were determined using the nonparametric Kruskal-Wallis test. (e) KEGG pathway enrichment analysis according to the markedly altered metabolites. The name of each KEGG pathway is shown on the left; the corresponding P-value is shown on the right and represented by a gradient color. P-values were determined using two-sided Fisher’s exact tests with Benjamini-Hochberg correction for multiple testing. (f) Random forest supervised machine-learning algorithm of metabolites in the H_1, H_2, D_1, and D_2 groups. The name of each metabolite is shown on the left. Top 10 metabolites in the fecal samples are shown in different colors; rank values are presented as MeanDecreaseGini. [file 40168_2022_1269_MOESM4_ESM.jpg]

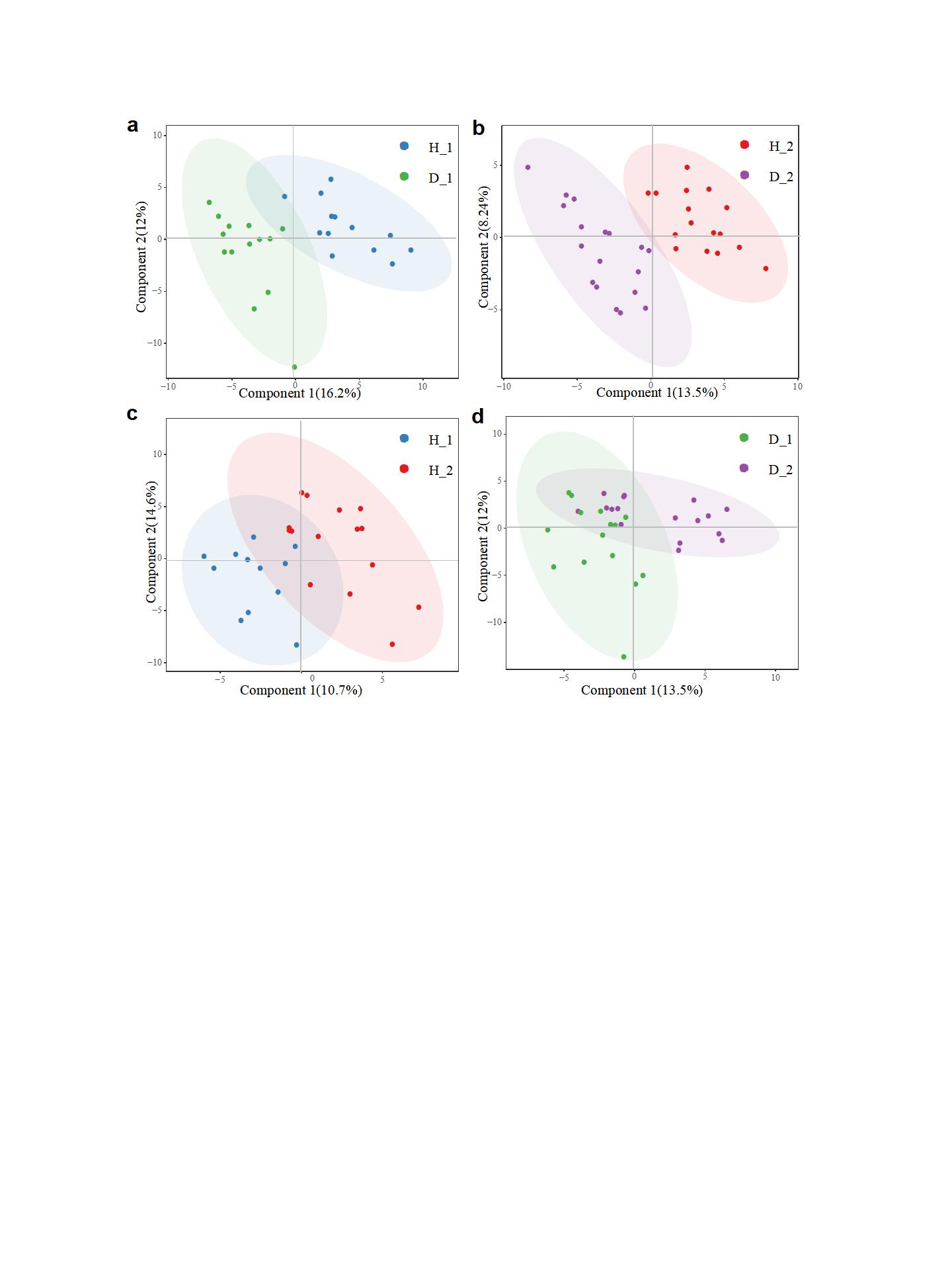

Supplement: Supplementary file 5 — Additional file 4: Fig. S4. Metabolomic analyses of fecal samples from healthy and diarrheic calves. Partial least squares discriminant analysis (PLS-DA) for dairy calves in H_1 vs D_1 (a), H_2 vs D_2 (b), H_1 vs H_2 (c), D_1 vs D_2 (d). H, healthy calves; D, diarrheic calves. [file 40168_2022_1269_MOESM5_ESM.jpg]

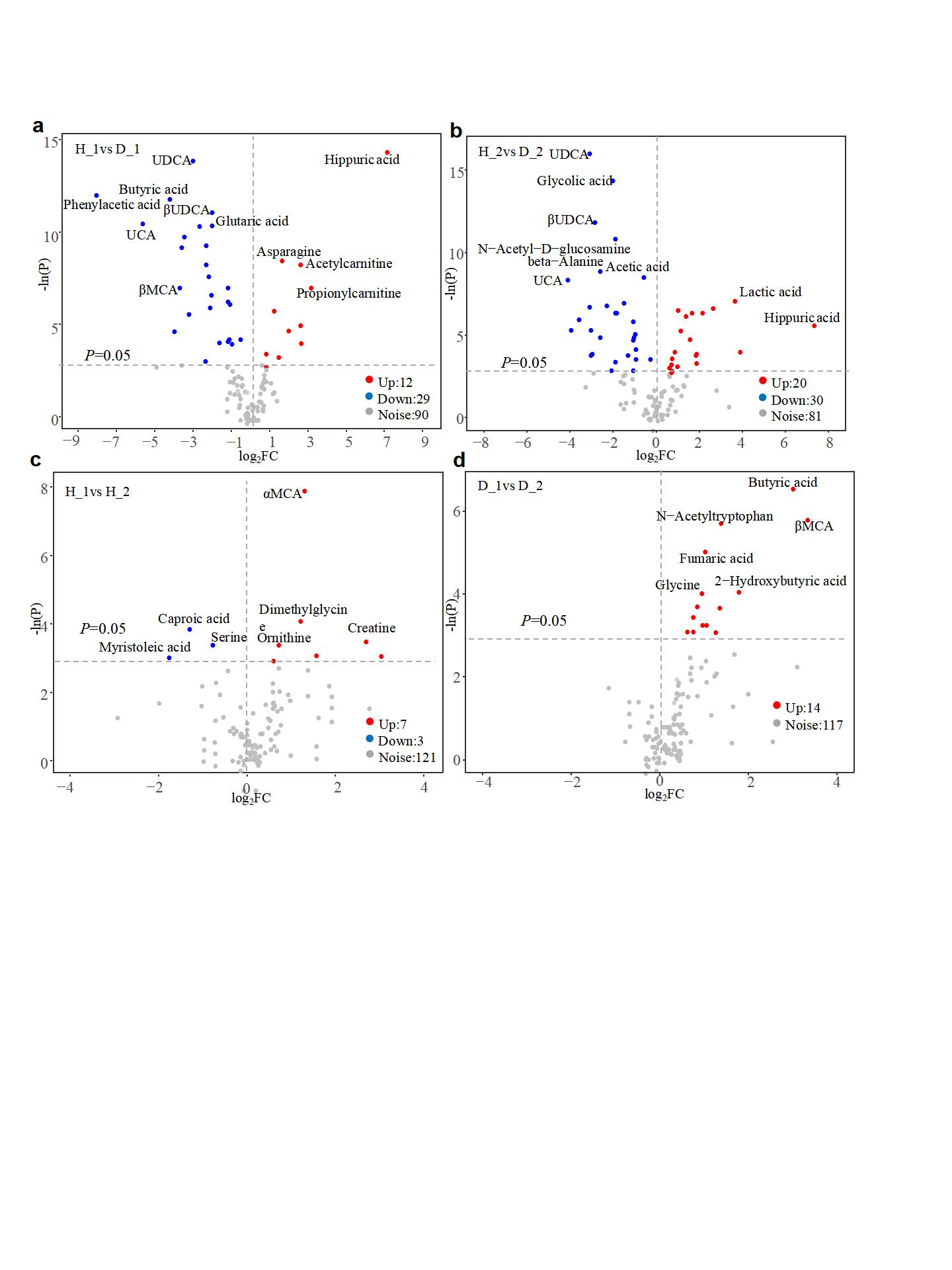

Supplement: Supplementary file 6 — Additional file 5: Fig. S5. Volcano maps of fecal metabolomics from healthy and diarrheic calves. Enriched metabolites were identified by analyses with P-values <0.05 and fold change (FC) values ≥1 (| log2FC|≥0). Significantly upregulated and downregulated metabolites are in red and blue, respectively. Metabolites with no obvious changes (noise) are gray. The differentiated metabolites in H_1 vs D_1 (a), H_2 vs D_2 (b), H_1 vs H_2 (c), D_1 vs D_2 (d) are shown. The numbers of upregulated and downregulated metabolites are on the right. [file 40168_2022_1269_MOESM6_ESM.jpg]

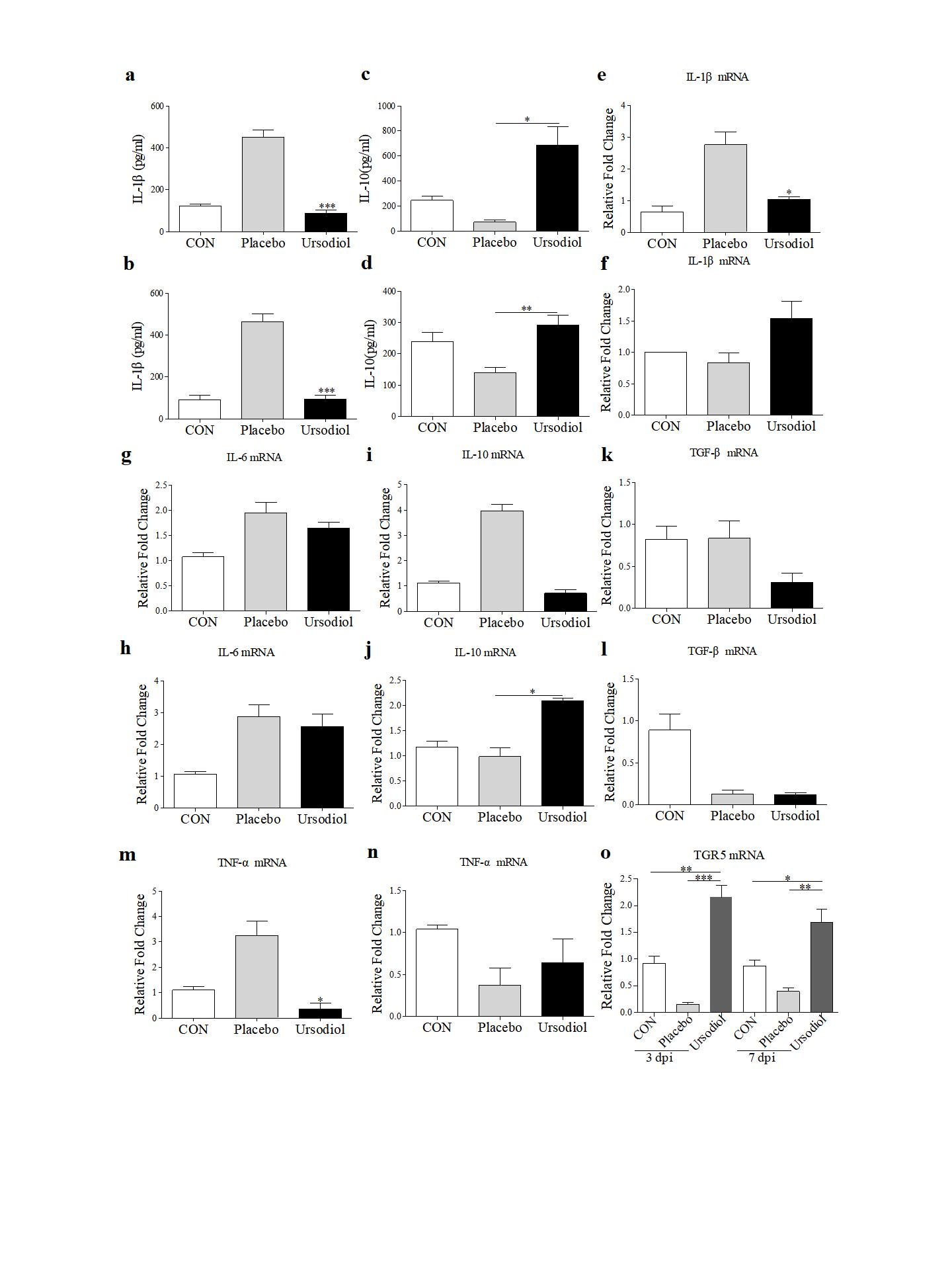

Supplement: Supplementary file 7 — Additional file 6: Fig. S6. Expression levels of inflammatory cytokines and TGR5 in a neonatal mouse model of peritonitis sepsis. IL-1β (a, b) and IL-10 (c, d) concentrations in the serum. Relative mRNA expression levels of five representative inflammatory cytokines, IL-1β (e, f), IL-6 (g, h), IL-10 (i, j), TGF-β (k, l), TNF-α (m, n), and TGR5 (o) in the colon. Data are presented as means±SEM. Statistical significance was analyzed using unpaired t-tests. *P≤0.05, **P≤0.01, ***P≤0.001. [file 40168_2022_1269_MOESM7_ESM.jpg]

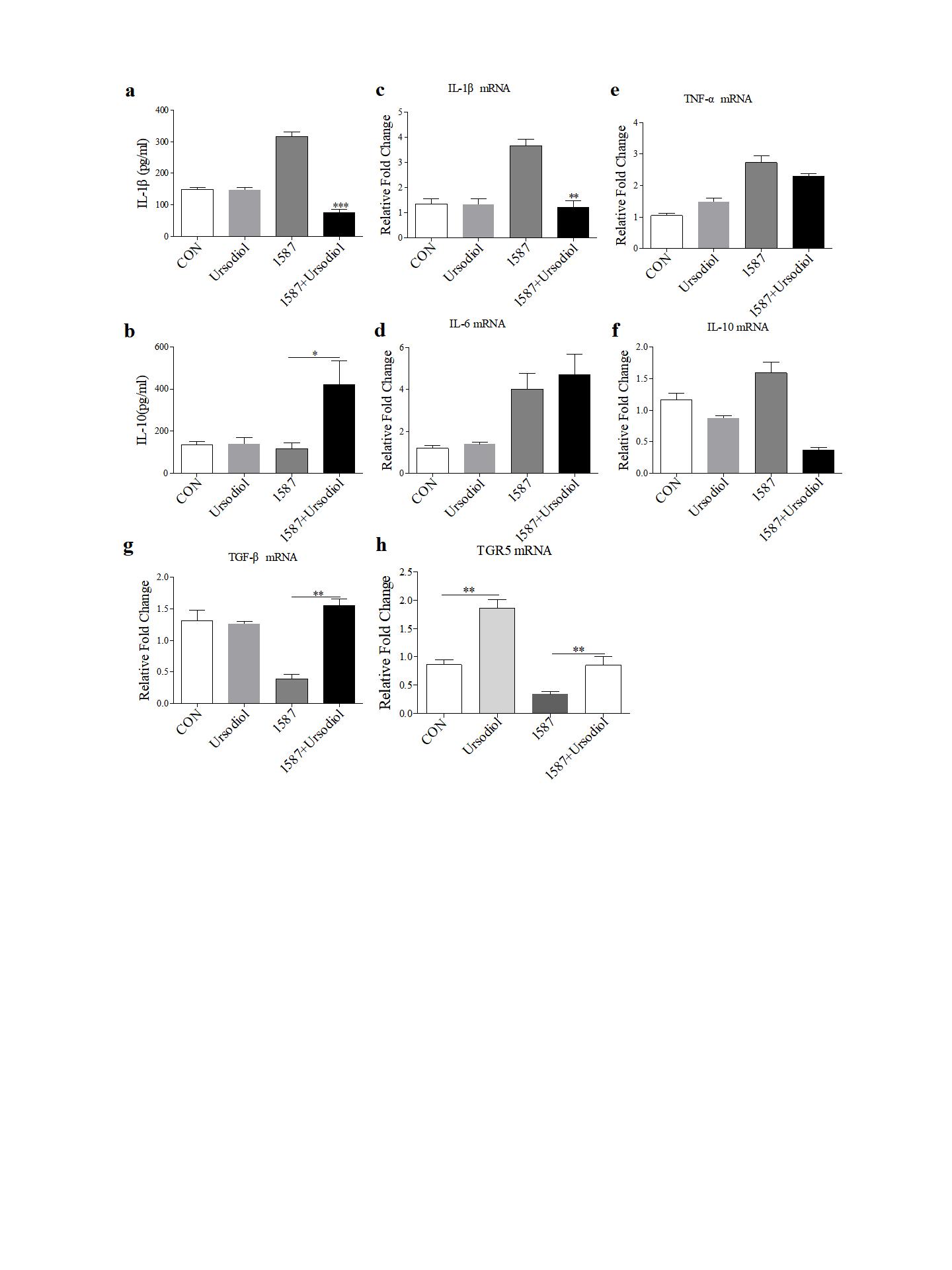

Supplement: Supplementary file 8 — Additional file 7: Fig. S7. Expression levels of inflammatory cytokines and TGR5 in neonatal mouse oral-infection model. IL-1β (a) and IL-10 (b) concentrations in the serum. Relative mRNA expression levels of five representative inflammatory cytokines, IL-1β (c), IL-6 (d), TNF-α (e), IL-10 (f), TGF-β (g), and TGR5 (h), in the colon. Data are presented as means±SEM. Statistical significance was analyzed using unpaired t-tests. *P≤0.05, **P≤0.01, ***P≤0.001. [file 40168_2022_1269_MOESM8_ESM.jpg]

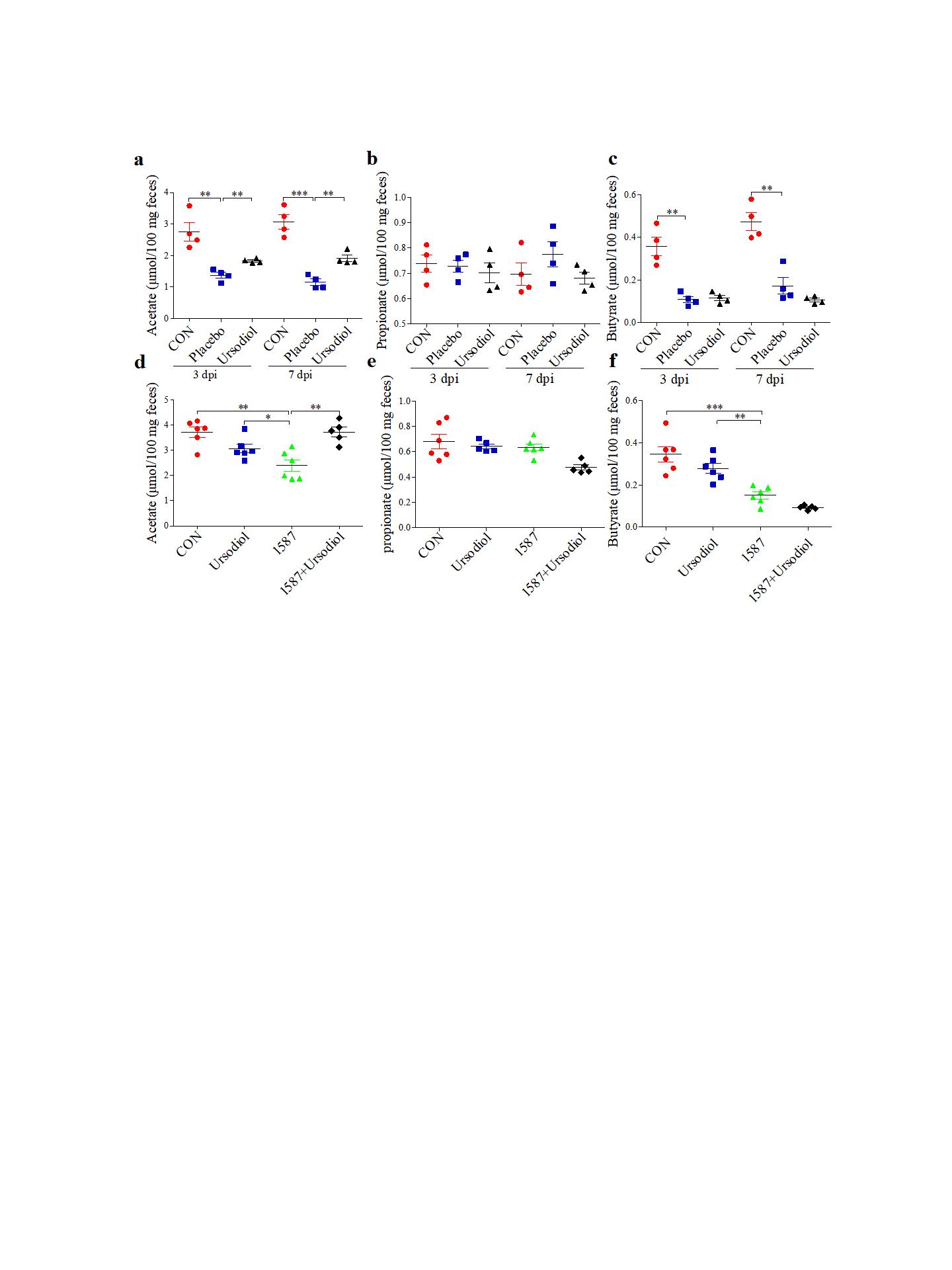

Supplement: Supplementary file 9 — Additional file 8: Fig. S8. SCFA concentrations in colonic contents after oral therapy. Concentrations of acetate (a, d), propionate (b, e), and butyrate (c, f) upon oral therapy (n = 4–6 per group). Data are presented as means±SEM. Statistical significance was analyzed using unpaired t-tests. *P≤0.05, **P≤0.01, ***P≤0.001. [file 40168_2022_1269_MOESM9_ESM.jpg]

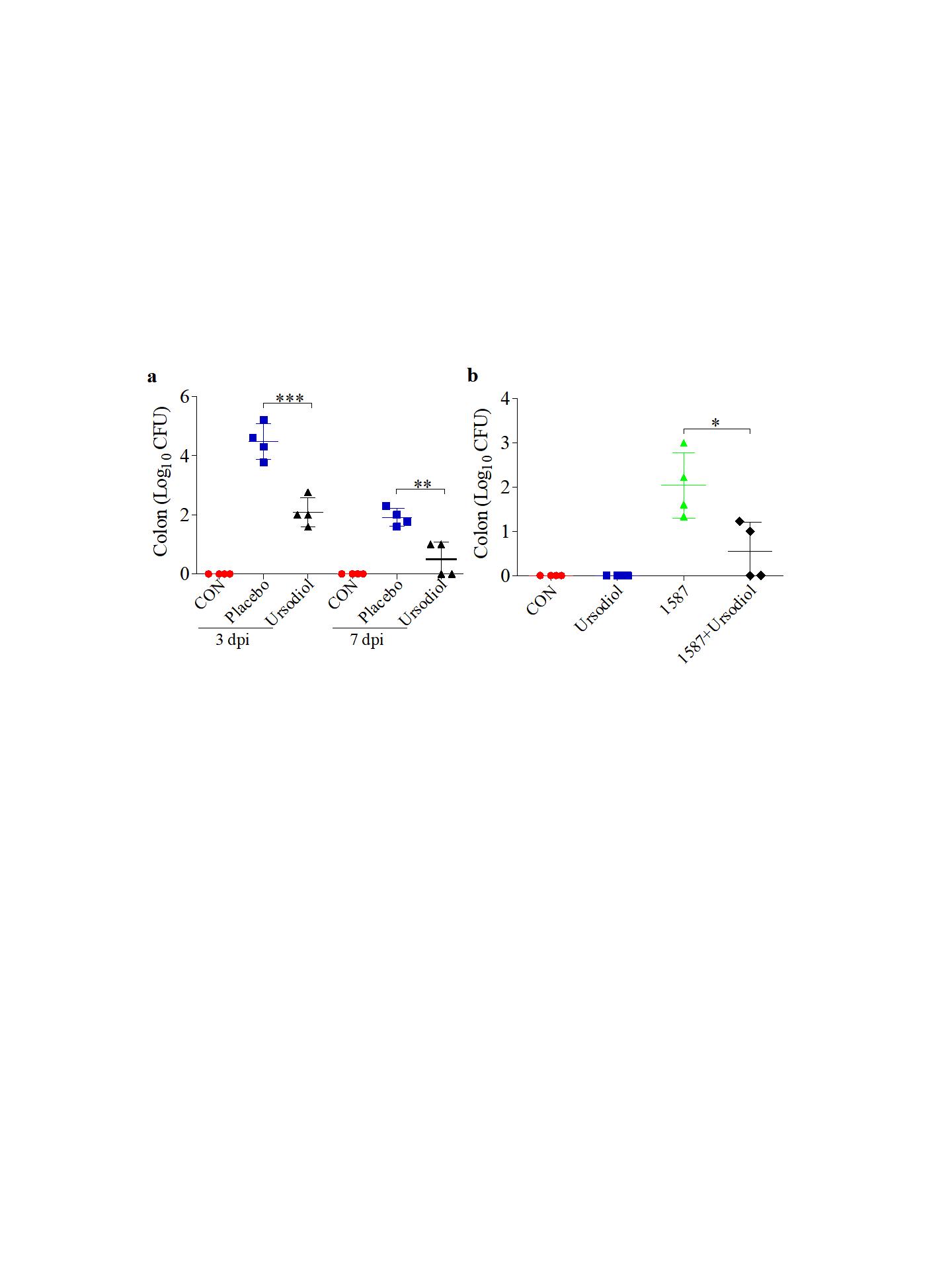

Supplement: Supplementary file 10 — Additional file 9: Fig. S9. Colonizations blocks of strain 1587 in colonic tissues of neonatal mouse infection models. Suppressive effect of oral ursodiol on 1587 strain colonization in neonatal mouse sepsis (a) and oral infection (b) models. Data are presented as means±SEM. Statistical significance was analyzed using unpaired t-tests. *P≤0.05, **P≤0.01, ***P≤0.001. [file 40168_2022_1269_MOESM10_ESM.jpg]

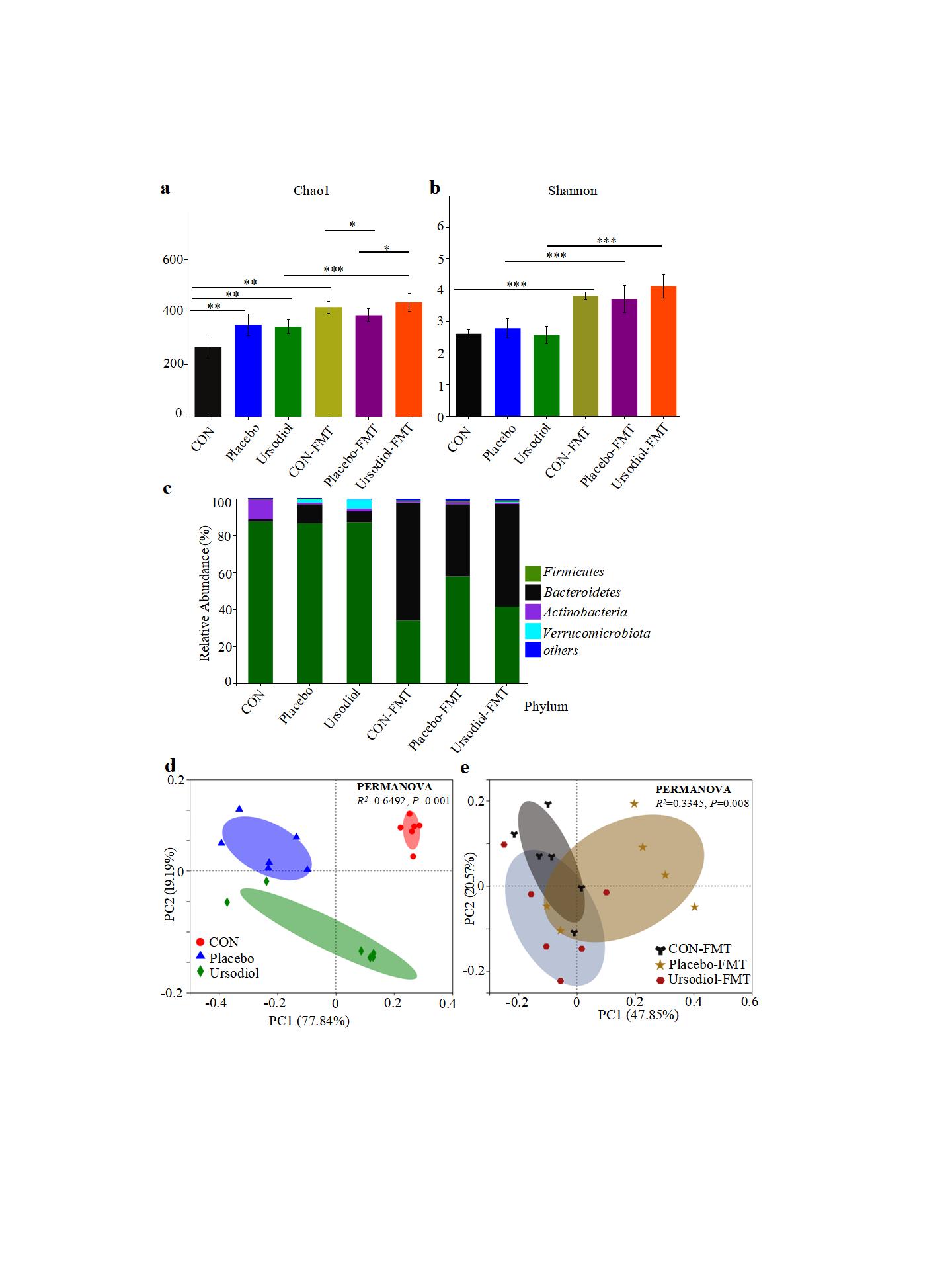

Supplement: Supplementary file 11 — Additional file 10: Fig. S10. Gut microbiota profiles of mouse donors and recipients. Alpha-diversity of different groups by Chao1 (a) or Shannon (b) index. Data are presented as the mean±SEM by unpaired t-test. *P≤0.05, **P≤0.01, ***P≤0.001. (c) Relative abundances of fecal bacterial phyla in 99.5% of the community. PCA plots based on the weighted UniFrac distance matrix of FMT donors (d; n = 6) and recipients (e; n = 6). Data were assessed using PERMANOVA analysis, with 999 permutations. [file 40168_2022_1269_MOESM11_ESM.jpg]

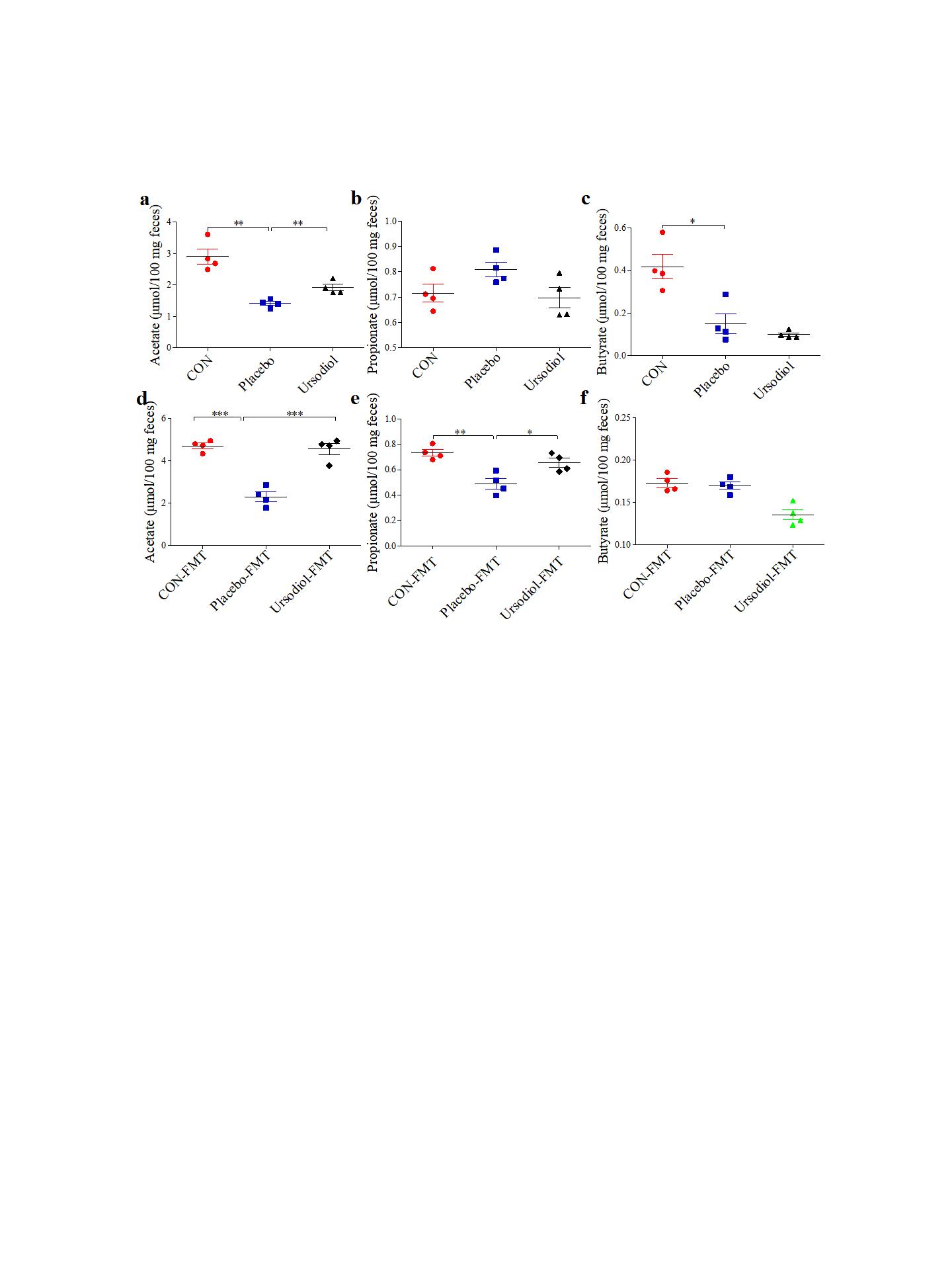

Supplement: Supplementary file 12 — Additional file 11: Fig. S11. SCFA concentrations in colonic contents of neonatal mouse donors and recipients. Concentrations of acetate (a), propionate (b), and butyrate (c) in donors upon oral therapy (n = 4 per group). Concentrations of acetate (d), propionate (e), and butyrate (f) in recipients upon FMT (n = 6 per group). Data are presented as means±SEM. Statistical significance was analyzed using unpaired t-tests. *P≤0.05, **P≤0.01, ***P≤0.001. [file 40168_2022_1269_MOESM12_ESM.jpg]

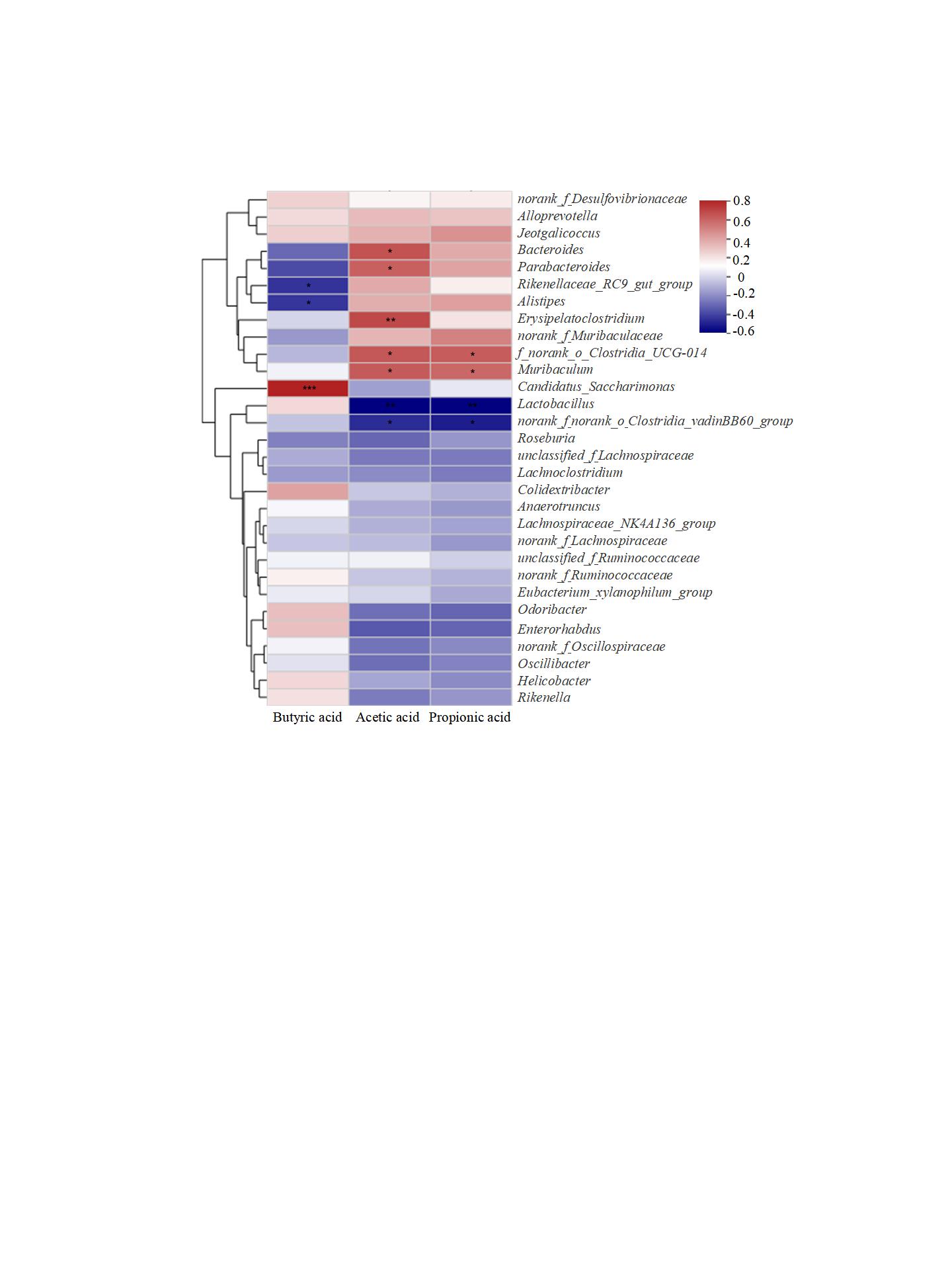

Supplement: Supplementary file 13 — Additional file 12: Fig. S12. Spearman correlation between the fecal microbiotas of FMT recipients and SCFA production of acetic, propionic, and butyric acid. Red denotes a positive correlation; blue denotes a negative correlation. Color intensity is proportional to the strength of the Spearman correlation. *P ≤ 0.05, **P≤0.01, ***P≤0.001. [file 40168_2022_1269_MOESM13_ESM.jpg]
